# Transcriptome, Proteome, and Metabolome Features in Diarrhea Challenge of Preweaning Piglets via Multi-Omics Integration Analyses

**DOI:** 10.3390/ani16111671

**Published:** 2026-05-29

**Authors:** Shilong Zhao, Siyi Peng, Guangxin Yang, Haitao Yu, Shiyan Qiao

**Affiliations:** State Key Laboratory of Animal Nutrition and Feeding, College of Animal Science and Technology, China Agricultural University, Beijing 100193, China; s20243040833@cau.edu.cn (S.Z.); ndpengsiyi@cau.edu.cn (S.P.); gx_yang2025@cau.edu.cn (G.Y.); qiaoshiyan@cau.edu.cn (S.Q.)

**Keywords:** preweaning piglets, multi-omics integration analysis, mitochondrial dysfunction, protein digestion and absorption, immune signaling pathways

## Abstract

Preweaning piglet diarrhea not only compromises animal health but also causes significant economic losses to the swine industry; however, the underlying molecular mechanisms remain poorly understood. Here, we use a preweaning piglet model to systematically explore the underlying risk features of pre-weaning diarrhea risk through multi-omics integration analysis. We identified thousands of differentially expressed genes, proteins, and metabolites. Key findings include: (1) activation of the complement cascade but downregulation of complement receptors; (2) increased chemokine production with reduced receptor expression; (3) overexpression of mitochondrial ribosomal proteins (MRPs) and NADH: ubiquinone oxidoreductase (NDUF), suggesting mitochondrial dysfunction; (4) aberrant activation of neuroactive ligand–receptor pathways; and (5) accumulation of undigested polypeptides in the jejunum due to reduced peptidase activity and amino acid transporter expression, indicating disrupted protein digestion and absorption. These findings provide critical insights and a valuable resource for addressing the underlying mechanisms of preweaning disease challenges in newborns.

## Simple Summary

Diarrhea is a major health threat to preweaning piglets, often leading to severe illness and even death. To gain a deeper understanding of the changes occurring in the intestines of piglets during diarrhea, this study employed advanced analytical techniques to investigate the genetic instructions, protein functions, and chemical reactions within the intestines of affected piglets. The results showed that diarrhea impairs the piglets’ ability to properly digest proteins, resulting in the accumulation of undigested protein fragments. At the same time, the immune system is activated in an ineffective manner, making the piglets more susceptible to further infections. Moreover, the study discovered that key energy-producing parts within the cells begin to malfunction, which may increase the risk of immune-related diseases. By uncovering these hidden issues, this research helps explain why diarrhea is so dangerous for young animals. These insights can guide the development of more effective prevention strategies and treatment methods, ultimately improving the survival rate and long-term health of newborn piglets and other young animals.

## 1. Introduction

The growth, development, and health status of preweaning piglets are critical determinants of swine performance and farm profitability across all production stages. Over the past two decades, the global pig farming industry has undergone significant advancements, yet disease challenges remain a persistent threat. Consequently, prioritising strategies to improve preweaning piglet survival is imperative. Notably, the intestinal systems of preweaning piglets are underdeveloped, and their gut microbial ecosystems are unstable. These physiological limitations render them highly susceptible to pathogens such as *Escherichia coli*, *Clostridium perfringens*, and porcine epidemic diarrhea virus (PEDV), which induce severe intestinal diseases, leading to reduced survival rates from birth to weaning and compromised post-weaning growth.

Among these pathogens, PEDV-induced porcine epidemic diarrhea (PED) is particularly devastating to preweaning piglets, causing acute diarrhea, dehydration, and stunted growth [1]. Mortality rates in PEDV-infected litters can approach 100% within days, whereas older pigs typically exhibit milder symptoms and recover spontaneously [2]. Current PED management relies on stringent biosecurity protocols and enhanced vaccination strategies [3]; however, no targeted therapies exist. Innovations in genetics, nutrition, and disease mitigation are thus essential to safeguard pork production and stabilise supply chains amid emerging biological threats.

Omics technologies—including transcriptomics, proteomics, and metabolomics—have emerged as transformative tools for understanding and managing livestock diseases [4]. These approaches enable comprehensive profiling of biochemical dynamics in animals, facilitating biomarker discovery, pathogen tracing, and preventive intervention design [5]. For instance, metabolomic studies have elucidated metabolic dysregulation in pigs infected with porcine reproductive and respiratory syndrome virus (PRRSV), advancing diagnostic applications [6]. Transcriptomic profiling identified HTRA3 and GFPT2 as key biomarkers distinguishing pigs resistant to African swine fever virus (ASFV) from susceptible individuals [7]. Similarly, proteomic analyses revealed that PEDV infection alters ileal protein abundance, with puerarin treatment restoring homeostasis [8]. Despite their potential, integrating multi-omics data (transcriptomic, proteomic, and metabolomic) remains challenging yet indispensable for unravelling complex host–pathogen interactions. However, integrating multi-omics data from the same individuals remains challenging due to technical and biological variability, and few studies have achieved a systematic cross-omics comparison in preweaning diarrhea.

This study aims to decode the molecular mechanisms underlying diarrhea in preweaning piglets through a multi-omics integration approach. We generated transcriptomic, proteomic, and metabolomic datasets from jejunal and colonic tissues of diarrheic and healthy piglets. By harmonising these data with advanced bioinformatics, we identified diarrhea-associated biomarkers, mapped cross-omics molecular networks, and inferred mechanistic pathways driving host responses. Our findings provide actionable insights to enhance piglet health management and sustainable swine production.

## 2. Materials and Methods

### 2.1. Studied Pigs and Sample Collection

The animals studied in this study were collected from the Fengning Pig Research Institute of China Agricultural University (Hebei, China). All the adult pigs lived in the same environment and were fed the same diet, and all the preweaning piglets were fed only breast milk. The daily diarrhea status of the piglets in the pigs was recorded until weaning (on the 20 days after birth). The piglets were divided into a diarrhea group (with persistent diarrhea since birth, defined by loose or unformed feces and perianal soiling observed for at least three consecutive days) and a healthy group (formed feces, normal demeanor, and no recent history of diarrhea). Twenty piglets from each group (a total of 40) were selected for euthanasia. The intestinal contents of these piglets were removed, and the remaining intestinal tissue was washed with physiological saline before collection. The jejunum and colon of each piglet were collected, and all samples were frozen in liquid nitrogen and stored at −80 °C for future use. After quality control, all 40 piglets were retained for each omics analysis (transcriptomics, proteomics, and metabolomics); no samples were excluded. The piglets were obtained from 10 multiparous sows (3–4 parity) with a litter size of 8–12 piglets per sow. Four piglets were selected from each sow (2 diarrheic and 2 healthy, where possible). Because individual sow identities and litter assignments were not recorded, litter effects were not considered in the statistical analyses. This limitation is addressed in the Section 4.

### 2.2. RNA Extraction and Transcriptomic Sequencing

Total RNA was extracted from each intestinal sample via TRIzol™ Reagent (Invitrogen, Carlsbad, CA, USA) following the manufacturer’s protocol. Following a quality check via agarose gel electrophoresis, the RNA quality was determined via a 5300 Bioanalyzer (Agilent, Santa Clara, CA, USA) and quantified via the ND-2000 (NanoDrop Technologies, Wilmington, DE, USA). For each intestinal sample, mRNA was enriched via oligo(dT) beads from 1 μg of total RNA. The sequencing libraries were subsequently constructed via the TruSeq™ RNA Sample Preparation Kit (Illumina, San Diego, CA, USA). This process involves a series of steps, including fragmentation, double-stranded cDNA synthesis, end-repair, phosphorylation, and adapter addition. The libraries were subsequently subjected to size selection and PCR amplification. Thereafter, sequencing was performed on an Illumina NovaSeq 6000 platform. Quality control for the raw reads was executed via fastp software (version 0.19.5) with the following parameters: <10% unknown nucleotides and >50% Q20 [9]. Mapping of the reference genome (Sscrofa11.1 in the Ensembl database) and gene expression calculations were performed via HISAT2 [10] and RSEM [11], respectively. Differential expression analysis was performed via DESeq2 [12] with the following criteria: a log2 (fold change) greater than 1 between the diarrhea and healthy samples and an FDR lower than 0.05. These genes were considered significantly differentially expressed genes (DEGs). The identification of the TFs within these DEGs was then facilitated by means of their annotation via AnimalTFDB 4.0 [13], a classification system that divides TFs into 73 families according to their DNA-binding domain.

### 2.3. Protein Extraction and Data-Independent Acquisition Mass Detection

The intestinal samples were suspended in protein lysis buffer (8 M urea, 1% SDS) with protease inhibitor, and then, the suspension was sonicated for 2 min and subjected to centrifugation at 12,000× *g* for 20 min at 4 °C to collect the proteins. The protein concentration was then measured via a BCA protein assay kit (Thermo Scientific, Waltham, MA, USA), and the quality of the extracted proteins was assessed via SDS–PAGE. Approximately 100 μg of protein from each sample was then resuspended in triethylammonium bicarbonate buffer (100 mM). The mixture was reduced with tris-(2-carboxyethyl)-phosphine (10 mM) for 1 h and alkylated with iodoacetamide (40 mM) for 40 min in darkness. The protein pellets were collected by centrifugation at 10,000× *g* at 4 °C for 20 min and then resuspended in 100 μL of triethylammonium bicarbonate buffer (100 mM). Trypsin was added to the suspension at a ratio of 1:50 trypsin-to-protein mass, and the mixture was then incubated at 37 °C for 18 h. Next, the peptides were dried via a vacuum pump and resolubilised with 0.1% trifluoroacetic acid. Thereafter, the peptides were desalted with HLB and dried again with a vacuum concentrator. Data-independent acquisition (DIA) proteomics was performed with a VanquishNeo (Thermo Fisher Scientific, Waltham, MA, USA) coupled with an Orbitrap Astral mass spectrometer (Thermo, USA) on the basis of the obtained peptides. Chromatographic separation was achieved via a PAC high-throughput column (75 μm × 5.5 cm, Thermo, USA) with a gradient of solvents A (water with 2% ACN and 0.1% formic acid) and B (water with 80% ACN and 0.1% formic acid). The chromatography run time was set to 8 min. The acquisition of DIA data was facilitated by an Orbitrap Astral mass spectrometer operating in DIA mode. The mass spectrometry scanning range was set between 100 and 1700 m/z. Spectronaut software (version 19) was then utilised to search the raw DIA data with the same reference genome used for transcriptomic sequencing to identify and assess the quality of the proteins. The fold change in protein expression between the diarrhea and healthy piglets was calculated, and the *p* value of the Wilcoxon rank-sum test was obtained via R. Thresholds of fold change (>1.2 or <0.83) and *p* value < 0.05 were utilised to identify differentially expressed proteins (DEPs).

### 2.4. Nontarget Metabolomics

The intestinal samples were individually ground with liquid nitrogen, and the homogenate was resuspended in prechilled 80% methanol. Following a five-minute incubation period, the mixture was subjected to a centrifugal process at 15,000× *g* at a temperature of 4 °C for 20 min. Thereafter, the upper layer was introduced into the LC–MS/MS system. LC analysis was performed on an Ultimate 3000 UHPLC System (Thermo Fisher Scientific, USA) coupled with an ACQUITY UPLC^®^ HSS T3 (150 × 2.1 mm, 1.8 µm; Waters, Milford, MA, USA). The detailed process for LC analysis was consistent with that used in a previous study [14]. The mass spectrometric detection of metabolites was performed on a Q Exactive instrument (Thermo Fisher Scientific, USA) with an ESI ion source. The MS detection parameters were selected in accordance with a previous report by Want [15]. The raw MS spectra were converted to mzXML format via Proteowizard software v3.0.8789 [16] and then quality controlled via the “XCMS” package [17] in R v4.2.2. The metabolites were identified by accurate mass and MS/MS data, which were subsequently matched with HMDB [18], MassBank [19], LipidMaps [20], and KEGG [21] data. The identification of differentially abundant metabolites (DAMs) between diarrheic and healthy piglets was achieved via the following threshold values: variable importance in projection (VIP) of the orthogonal partial least square discrimination analysis > 1, |log2(fold change) | > 1, and false discovery rate (FDR)-adjusted *p* value < 0.05.

### 2.5. Statistical Analysis

All the statistical analyses were conducted via the R v4.2.2 platform, and the results were visualised via the ggplot2 [22] package.

#### 2.5.1. Multivariate Analysis (Principal Component Analysis and Adonis Test)

Principal component analysis (PCA) was performed via the “FactoMineR” package (version 2.14) [23] to assess variations in gene expression profiles, protein expression profiles and metabolite compositions between samples from different intestinal sections and different health statuses. Furthermore, the adonis test was conducted via the “vegan” package (version 2.7-1) [24] to evaluate the significance of the effects of intestinal sections and diarrhea on the variations in these biochemicals.

#### 2.5.2. Differential Expression and Functional Enrichment Analysis

The KEGG pathways associated with the DEGs and DEPs were then visualised via the iPath v3 web tool (http://pathways2.embl.de) (accessed on 17 January 2026). Furthermore, the KEGG annotations of the DEGs and DEPs were obtained from the reference genome, and functional enrichment analysis was carried out via clusterProfiler 4.0 [25]. The KEGG enrichment analysis of the DAMs was performed via MetaboAnalyst software (version 6.0) [26].

#### 2.5.3. Network Analysis

The protein–protein interaction (PPI) associations of the identified DEGs and DEPs were obtained from the STRING database to construct a protein–protein interaction (PPI) network [27]. Furthermore, weighted gene coexpression network analysis (WGCNA) was performed on the DEGs identified from the jejunum and colon samples to identify potential key gene modules in response to diarrhea via the “WGCNA” package (version 1.73) [28].

#### 2.5.4. Correlation Analysis Between Transcriptome and Proteome

The Mantel test and Procrustes analysis were utilised to evaluate the correlations between the gene and protein expression profiles (“vegan” package). A Venn diagram was constructed via the “VennDiagram” package (version 1.8.0) [29] to confirm the presence of the detected genes in the transcribed genome that contained the coding genes of the detected proteins from the proteome. Consequently, nine-quadrant diagrams were constructed on the basis of these consistent proteins and their coding genes to indicate the correlation between the transcriptome and the proteome. The shared DEPs and their coding genes (DEG/Ps) that were consistently up- or downregulated in the same intestinal section were also recognised via a Venn diagram. Subsequently, correlation networks of shared DEGs/Ps and DAMs were constructed for the jejunum and colon, respectively. Spearman’s rank correlations were then calculated among the expression levels of shared DEGs/Ps and the abundance of DAMs, with only those correlations exhibiting a |correlation coefficient| > 0.8 and a *p* value < 0.05 being retained for further analysis. The topological roles of individual nodes in networks were evaluated by the threshold values of Pi (measuring how well a node was connected to nodes in different modules) and Zi (measuring how well a node was connected to other nodes in its own module), which were calculated via the “igraph” package (version 2.2.0) [30]. The nodes can then be defined as follows: network hubs (Zi > 2.5; Pi > 0.6), module hubs (Zi > 2.5; Pi < 0.6), connectors (Zi < 2.5; Pi > 0.6) and peripherals (Zi < 2.5; Pi < 0.6) [31].

#### 2.5.5. Multi-Omics Integration

Multiple coinertia analysis (MCIA) was employed to identify correlations between the expression profiles of differentially expressed genes (DEGs) and differentially expressed proteins (DEPs), as well as between the DEG/P expression profiles and the abundance compositions of differentially abundant metabolites (DAMs) [32]. By utilising a covariance optimisation criterion, the MCIA extracts the most variant from each dataset, and their correlations are analysed via Spearman’s rank correlation. Furthermore, multiomics factor analysis (MOFA) was applied to the same datasets of MCIA to discover the principal sources of variation in multiomics datasets [33]. By inferring a set of factors that capture biological and technical sources of variability via MOFA, learned factors that contribute to distinct diarrhea and healthy samples were identified. The top features loaded on these significant factors were subsequently obtained, and their correlations were also assessed via Spearman’s rank correlation.

## 3. Results

### 3.1. Variations in Gene Expression Profiles

**Figure 1:**
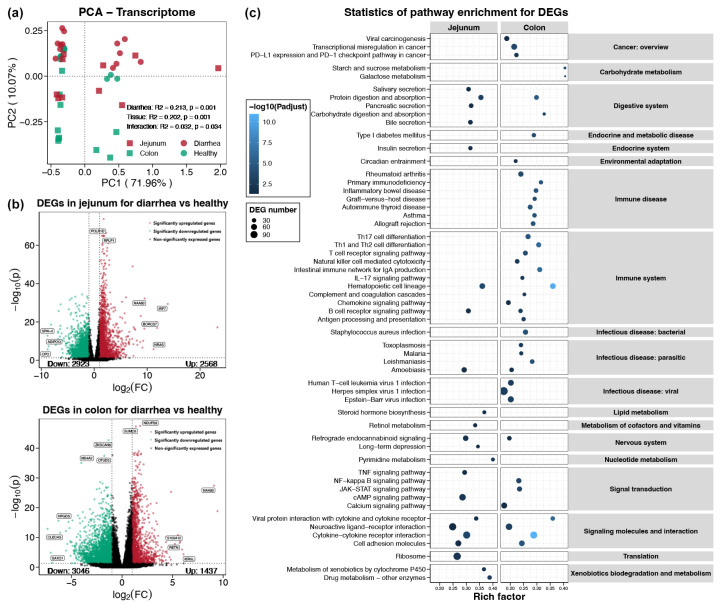
Comparisons of the gene expression profiles of the jejunum and colon, respectively, between the diarrheic and healthy piglets. (**a**) Principal component analysis (PCA) and Adonis test showing the variations in gene expression profiles between different health status and intestinal sections of piglets. (**b**) Volcano plots exhibiting the differentially expressed genes (DEGs) between the diarrhea and healthy piglets in their jejunum and colon, respectively. (**c**) Bubble chart that displays the results of KEGG enrichment analysis based on the DEGs recognized from the jejunum and colon samples, respectively.

A total of 1 billion reads were obtained from the RNA-Seq data of 40 samples, with more than 70% of these mapped to the coding sequence of the reference genome. This analysis revealed a total of 27,100 transcripts. PCA revealed that the gene expression profiles of the diarrheic and healthy piglets were separated by the PC1 axis, which explained 71.96% of the total variation (Figure 1a). Conversely, samples from the jejunum and colon were distributed along the PC2 axis, which explained 10.07% of the total variation (Figure 1a). The Adonis test indicated significant impacts of diarrhea and intestinal section on the gene expression profiles of piglets (*p* < 0.05), which tended to play an independent role (R^2^ = 0.213 and 0.202, respectively, for each alone compared with 0.032 for their interaction, Figure 1a). In the jejunum section, 2568 and 2923 differentially expressed genes (DEGs) were identified with significantly up- and downregulated expression in diarrhea samples compared with healthy piglets, respectively (Figure 1b). Among these genes, the genes IRF7, BORCS7, NRAS, NAA80, POLRD, and RPLP1 presented the most significant upregulation, whereas SPAI-2, ADIPOQ, and CPO presented the most pronounced downregulation (Figure 1b). A total of 1437 upregulated differentially expressed genes (DEGs) were identified in the diarrhoea colon samples compared with their healthy counterparts, with representatives of NAA80, S100A121, RETN, IER5L, NDUFS8, and SUMO3 (Figure 1b). Conversely, 3046 genes with ZKSCAN8, MS4A2, OTUD3, HPGDS, CLEC4G, and SAXO1 expression were significantly downregulated in the diarrheic colon samples compared with the healthy samples (Figure 1b).

In both the jejunum and the colon, the DEGs were predominantly annotated to perform functions related to signal transduction, the immune system and viral infections (Figures S1 and S2). Furthermore, 146 DEGs in the jejunum were associated with translation; however, no DEGs related to this function were observed in the colon samples (Figure S2). The KEGG enrichment analysis of the DEGs revealed pathways that were enriched in both the jejunum and colon of piglets. These pathways included protein digestion and absorption, the hematopoietic cell lineage, the B-cell receptor signalling pathway, amoebiasis, retrograde endocannabinoid signalling, and four signalling molecules and interaction pathways (Figure 1c). Furthermore, a number of pathways related to diarrhea were enriched in the jejunum or colon of piglets. The enriched pathways in the jejunum were predominantly associated with xenobiotic metabolism, translation, pyrimidine metabolism, retinol metabolism, and hormone secretion (Figure 1c). Conversely, the colon was enriched in pathways related to infectious disease, carbohydrate metabolism, the immune system, and disease infections (Figure 1c). The differential enrichment of these pathways may indicate distinct functions of the jejunum and the colon in the context of piglet diarrhea. The diarrhea-related phenotype may occur primarily in the colon, whereas the changes in jejunal gene expression might be related to hormone secretion.

### 3.2. Gene Expression Modules Recognization

**Figure 2:**
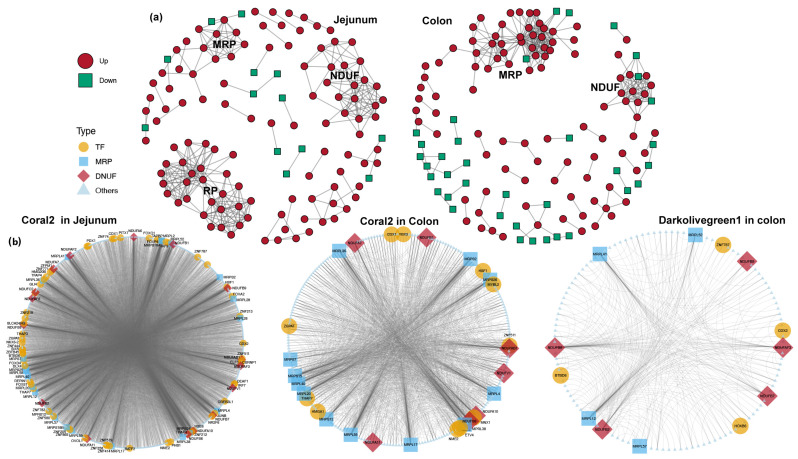
The identification of key gene modules associated with diarrhoea in piglets. (**a**) The protein–protein interaction (PPI) networks are based on the DEGs in the jejunum and colon, respectively. (**b**) Gene expression modules related to the NDUF (NADH: ubiquinone oxidoreductase) and MRP (mitochondrial ribosomal protein) gene families recognized by WGCNA based on the DEGs in jejunum and colon, respectively.

The PPI networks of DEGs in the jejunum and colon were constructed on the basis of prior knowledge (Figure 2a). Three upregulated interaction modules were identified in the jejunum: (i) a module comprising ribosomal proteins (RBs) and including diverse RPL and RPS genes; (ii) a module comprising mitochondrial ribosomal proteins (MRPs) and including multiple MRPL and MRPS genes; and (iii) a module comprising NADH: ubiquinone oxidoreductase (NDUF). Concurrently, the MRP and NDUF modules were identified as being upregulated in the PPI network of the colon, with the MRP module exhibiting a greater number of members and the NDUF module containing a smaller number of genes. The subsequent identification of TFs among the DEGs revealed that the zf-C2H2, homeobox, ZBTB, bHLH, TF_bZIP, ETS, and HMG families were the predominant TF families (Figure S3). Among these families, the zf-C2H2, ZBTB, and HMG families presented a greater number of upregulated DEGs, whereas the ETS and TF_bZIP families presented a greater proportion of downregulated DEGs (Figure S3). Furthermore, WGCNA was performed to identify key gene modules related to diarrhea in piglets. A total of 26 and 13 gene expression modules were identified from the DEGs in the jejunum and colon, respectively (Figure S4). Among these genes, one module (coral2) in the jejunum was found to contain 16 NDUF genes and 20 MRP genes in conjunction with a variety of TFs (Figure 2b). In the colon, two modules were identified as being associated with the NDUF and MRP gene families: the coral1 module (7 NDUF, 12 MRP, and 11 TFs) and the darkolivegreen1 module (5 NDUF, 4 MRP, and 4 TFs) (Figure 2b). These results indicated that the upregulated expression of the NDUF and MRP genes could play a vital role in diarrhea in piglets.

### 3.3. Variations in Protein Expression Profiles

**Figure 3:**
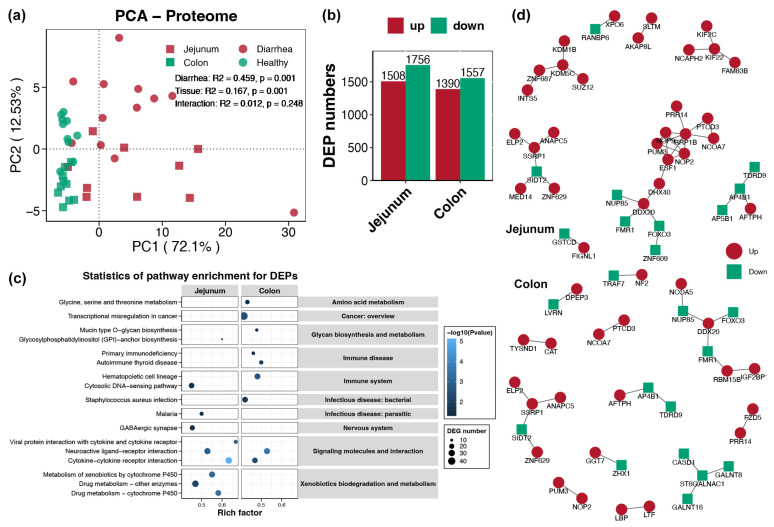
Comparisons of the protein expression profiles of the jejunum and colon, respectively, between the diarrheic and healthy piglets. (**a**) Principal component analysis (PCA) and Adonis test showing the variations in protein expression profiles between different health status and intestinal sections of piglets. (**b**) Volcano plots exhibiting the differentially expressed proteins (DEPs) between the diarrhea and healthy piglets in their jejunum and colon, respectively. (**c**) A bubble chart that displays the results of KEGG enrichment analysis based on the DEPs recognised from the jejunum and colon samples, respectively. (**d**) The protein–protein interaction (PPI) networks based on the DEPs in jejunum and colon, respectively.

A total of 150,000 peptides were identified from the DIA proteome dataset, which is attributable to 9843 proteins. In a manner analogous to the results of the gene expression profiles, PCA also revealed that the protein expression profiles of diarrheic and healthy piglets were separated by the PC1 axis and that samples from the jejunum and colon were distributed along the PC2 axis (Figure 3a). Furthermore, the adonis test yielded significant independent impacts of diarrhea and intestinal sections on the protein expression profiles (*p* < 0.05). In contrast to the comparable contributions of diarrhea and intestinal sections to variations in gene expression profiles (0.213 vs. 0.202, Figure 1a), diarrhea had a more pronounced effect on protein expression (R^2^ = 0.459) than did intestinal section (R^2^ = 0.167) (Figure 3a). In the jejunum, 1508 proteins were found to be upregulated in diarrheic piglets compared with their healthy counterparts, and 1756 proteins were downregulated (Figure 3b). For the colon, a total of 1390 and 1557 up- and downregulated DEPs, respectively, were identified in samples from diarrheic subjects compared with those from healthy individuals (Figure 3b). Like the functions of the DEGs, the functions of the DEPs were also associated primarily with signal transduction, the immune system, and infectious diseases (Figures S5 and S6). In the jejunum, 98 DEPs related to lipid metabolism were unique, whereas 76 specific DEPs associated with environmental adaptation were detected in the colon (Figure S6). While the number of enriched KEGG pathways related to the DEPs was considerably lower than that related to the DEGs, pathways related to signalling molecules and interactions were found to be the most enriched pathways in both the jejunum and the colon (Figure 3c). In a manner analogous to the results obtained for the DEGs (Figure 1c), pathways associated with xenobiotic metabolism and pathways linked to the immune system and disease infections were also enriched in the jejunum and colon, respectively, according to the DEPs (Figure 3c). The PPI networks were also constructed on the basis of the DEPs, while no obvious module was identified, such as the PPI networks based on the DEGs. Furthermore, analysis of the jejunum and colon DEP networks revealed the presence of protein interactions that were centralised by the DEAD-Box helicase 20 (DDX20) protein (Figure 3d).

### 3.4. Correlations of Gene and Protein Expression Profiles

The Venn diagram indicated that the coding genes of all the detected proteins based on the proteomics data were identified via RNA-Seq (Figure S7a). Furthermore, the Mantel test and Procrustes analysis indicated a high degree of consistency between the gene and protein expression profiles across all the samples studied (*p* < 0.05, Figure S7b). Furthermore, the nine-quadrant diagrams revealed a significant correlation between the expression of proteins and their coding genes in both the jejunum and the colon (Pearson correlation, *p* < 0.05; Figure S7c,d). These results support the reliability of the omics data and suggest that joint analysis of the transcriptome and proteome might provide a more accurate exploration of the underlying mechanisms of piglet diarrhea.

**Figure 4:**
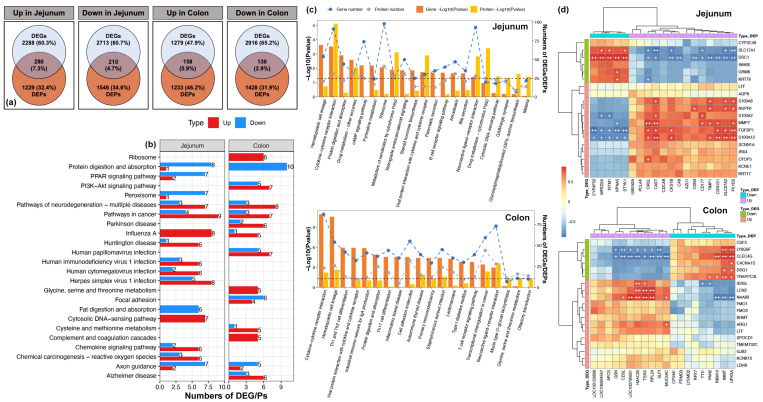
Correlations of the gene and protein expression profiles in the jejunum and colon of piglets. (**a**) Venn diagrams illustrating the shared DEPs and their coding genes that were consistently up- or down-regulated expressed in jejunum and colon samples, respectively. (**b**) The top 15 KEGG pathways with the highest number of shared DEGs/Ps (DEG/P: differentially expressed gene/protein) identified in the jejunum and colon, respectively. The value adjacent each bar represents the number of shared DEG/Ps belonging to the corresponding pathway. (**c**) Common pathways of KEGG enrichment analysis of DEGs and DEPs between diarrhea and healthy piglets in jejunum and colon samples, respectively. (**d**) The following heatmaps reveal the correlations of expression levels of top 20 key DEGs and DEPs to reflect the variations in gene and protein profiles identified by the MCIA. The colour of each block corresponds to Spearman’s correlation coefficient, with a + symbol indicating a *p*-value less than 0.05 and ++ indicating a *p*-value less than 0.01.

In the jejunum, a total of 280 and 210 up- and downregulated shared DEG-DEPs (DEG/Ps) were identified, respectively (Figure 4a). Concurrently, 158 and 130 up- and downregulated shared DEGs/Ps were identified in the colon, respectively (Figure 4a). The functions of these shared DEGs/Ps included nutrient metabolism (protein and fat), signal transduction (PPAR, PI3K-Akt, and chemokine), immune diseases (focal adhesion, complement cascades, and several diseases), and virus infections (Figure 4b). Furthermore, DEGs/Ps associated with nutrient metabolism were predominantly downregulated in piglets with diarrhea, whereas those associated with immune diseases and virus infections were predominantly upregulated (Figure 4b). Furthermore, a number of pathways, including cytokine–cytokine receptor interaction, drug metabolism–other enzymes, metabolism of xenobiotics by cytochrome P450, viral protein interaction with cytokines and cytokine receptors, and neuroactive ligand–receptor interaction in the jejunum, were found to be significantly enriched in piglets with diarrhea on the basis of both DEGs and DEPs. Furthermore, cytokine–cytokine receptor interaction, hematopoietic cell lineage, autoimmune thyroid disease, primary immunodeficiency, Staphylococcus aureus infection, transcriptional misregulation in immunosuppression, and neuroactive ligand–receptor interaction in the colon were found to be significantly enriched (Figure 4c). Finally, potential key genes and proteins for revealing variations in gene and protein expression profiles were identified via MCIA. The first two components accounted for more than 50% of the variation in gene expression profiles and 70% of the variation in protein expression profiles in both the jejunum and the colon (Figure S8). The key genes recognised in the jejunum were closely correlated with certain proteins, including solute carrier family 17 member 1 (SLC17A1), desmocollin 1 (DSC1), matrix metallopeptidase 7 (MMP7), fibroblast growth factor binding protein 1 (FGFBP1), and S100 calcium binding protein A12 (S100A12) (Figure 4d). In the colon, the identified key genes were predominantly associated with proteins belonging to the lymphocyte antigen 6 family member G6F (LY6G6F), C-type lectin domain family 4 member G (CLEC4G), and N-alpha-acetyltransferase 80 (NAA80) (Figure 4d).

### 3.5. Variations in Metabolite Compositions

**Figure 5:**
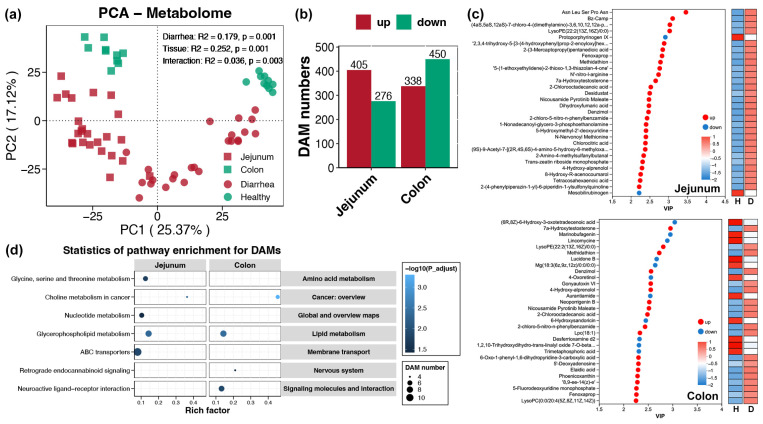
Comparisons of the metabolite compositions of the jejunum and colon, respectively, between the diarrheic and healthy piglets. (**a**) Principal component analysis (PCA) and Adonis test showing the variations in metabolite compositions between different health status and intestinal sections of piglets. (**b**) Volcano plots exhibiting the differentially abundant metabolites (DAMs) between the diarrhea and healthy piglets in their jejunum and colon, respectively. (**c**) Variable importance in projection (VIP) plots showing the DAMs with maximum abundance variations in the jejunum and colon between diarrhea and healthy piglets. The full names of the compounds indicated by the ellipses (from top to bottom) are: (4aS,5aS,12aS)-7-chloro-4-(dimethylamino)-3,6,10,12,12a-pentahydroxy-6-methyl-1,11-dioxo-1,4,4a,5,5a,6,11,12a-octahydrotetracene-2-carboxamide; ‘2,3,4-trihydroxy-5-[3-(4-hydroxyphenyl)prop-2-enoyloxy]hexanoic acid; (9S)-9-Acetyl-7-[(2R,4S,6S)-4-amino-5-hydroxy-6-methyloxan-2-yl]oxy-6,9,11-trihydroxy-8,10-dihydro-7H-tetracene-5,12-dione; 1,2,10-Trihydroxydihydro-*trans*-linalyl oxide 7-O-beta-D-glucopyranoside. (**d**) A bubble chart is employed to display the results of KEGG enrichment analysis based on the DAMs recognized from the jejunum and colon samples, respectively.

A total of 2715 metabolites were identified from the LC–MS untargeted metabolome dataset of 40 samples. The application of PCA to the metabolite compositions resulted in the identification of separate clusters of samples from different intestinal sections and different health statuses (Figure 5a). In contrast to the more pronounced impact of diarrhea on gene and protein expression profiles, the influence of diarrhea (R^2^ = 0.179) on metabolite compositions was comparatively weaker than that of the intestinal section (R^2^ = 0.252) (Adonis test, *p* < 0.05). A total of 405 metabolites were found to be significantly more abundant in the jejunum affected by diarrhea than in the healthy jejunum, while 276 metabolites were found to be downregulated (Figure 5b). Compared with their healthy counterparts, 338 and 450 up- and downregulated DAMs were identified in the diarrheic colon (Figure 5b). Among these DAMs, phospholipids were the most prevalent class in both the jejunum and the colon, with multiple eicosanoids being identified in the jejunum (Figure S9). The functions of these DAMs are associated primarily with metabolism, particularly lipid metabolism, and infectious diseases (Figure S10). In the jejunum, pentapeptides (Asn-Leu-Ser-Pro-Asn) and LysoPE (lysophosphatidylethanolamine, 22:2(13Z, 16Z/0:0)) were most enriched in diarrheic piglets, whereas protoporphyrinogen IX and mesobilirubinogen were most reduced (Figure 5c). In the colon, the metabolites LysoPE (22:2(13Z,16Z/0:0)) and mesobilirubinogen were also increased and decreased, respectively, in diarrheic piglets (Figure 5c). On the basis of these DAMs, pathways related to choline metabolism in immune-related diseases and glycerophospholipid metabolism were enriched in both the jejunum and colon of piglets (Figure 5d). Furthermore, enrichment was observed in glycine, serine, and threonine metabolism; nucleotide metabolism; and ABC transporters in the jejunum and in retrograde endocannabinoid signalling and neuroactive ligand–receptor interaction pathways in the colon (Figure 5d).

### 3.6. Associations Between Shared DEGs/Ps and DAMs

**Figure 6:**
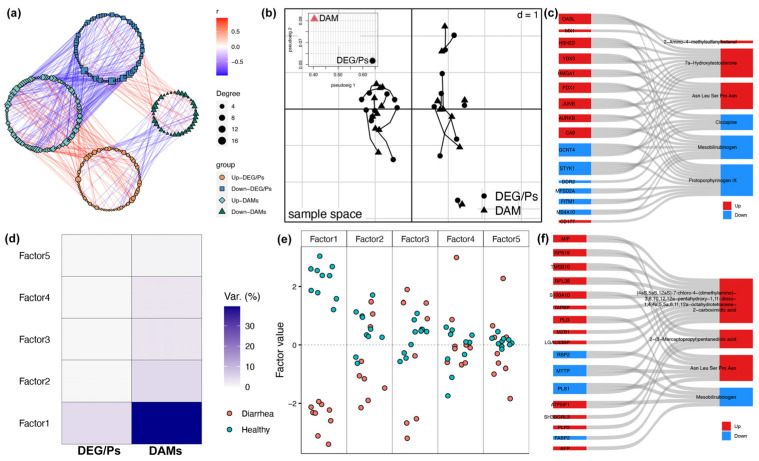
The associations between shared DEG/Ps and DAMs in the jejunum of piglets. (**a**) The correlation network of shared DEG/Ps and DAMs is based on Spearman’s correlation with *p*-value adjusted by the false detection ratio (FDR) method. It is important to note that only correlations that were found to be statistically significant (|r| > 0.8 and FDR < 0.05) are displayed. (**b**) The subsequent analysis focuses on sample co-variation, underpinned by the MCIA and the eigenvalues of shared DEG/Ps and DAMs across the initial two pseudoeig axes. (**c**) The Sankey diagram is employed to illustrate the correlations between the expression levels of the top 20 important shared DEGs/Ps and the abundances of the top 20 important DAMs, as identified by the MCIA. It is important to note that only correlations that have been determined to be statistically significant (|r| > 0.8 and FDR < 0.05) are displayed by the grey connecting lines. (**d**) A heatmap is employed to illustrate the explanatory proportion of the top 5 integration factors obtained by the MOFA to the variations in shared DEG/Ps and DAMs. (**e**) The distribution of factor values of each sample among the top five integration factors obtained by the MOFA is revealed by the scatter plot. (**f**) A Sankey diagram is employed to exhibit the correlations between the expression levels of the top 20 important shared DEGs/Ps and the abundances of the top 20 important DAMs that have been identified by the MOFA. It is noteworthy that only correlations that have been determined to be statistically significant (|r| > 0.8 and FDR < 0.05) are displayed by the grey connecting lines.

To further explore the underlying molecular variations behind diarrhea in piglets, the associations between shared DEGs/Ps and DAMs in the jejunum and colon were investigated. In the jejunum, 100 links between shared DEGs/Ps and DAMs were identified in the correlation network (Figure 6a and Figure S11), but no keystone genes were identified (Figure S11c). The first two components of the MCIA explained 73% and 51% of the variation in the number of shared DEGs/Ps and DAMs, respectively (Figure 6b). The top 20 important DEGs/Ps and DAMs identified in the MCIA were then used to identify multiple key DEGs/Ps that were significantly correlated with 7a-hydroxytestosterone, Asn-Leu-Ser-Pro-Asn, clozapine, mesobilirubinogen, and protoporphyrinogen IX (Spearman correlation, *p* < 0.05; Figure 6c). The MOFA method was further applied to identify key integration factors that explain differences between samples, and the results revealed that the top 5 factors explained 48.15% of the DAM changes but explained only 4.36% of the shared DEG/Ps variations (Figure 6d). Utilising the factor values of diverse samples, the top 2 factors were found to be capable of differentiating jejunum samples exhibiting distinct health statuses (Figure 6e), and the key DEG/Ps and DAMs for these factors were identified (Figures S12 and S13). Among these biomarkers, the majority of the DEGs/Ps presented significant correlations with four DAMs (2-carboximidic acid derivant, 2-(3-mercaptopropyl) pentanedioic acid, Asn-Leu-Ser-Pro-Asn, and mesobilirubinogen) (Spearman correlation, *p* < 0.05; Figure 6f).

**Figure 7:**
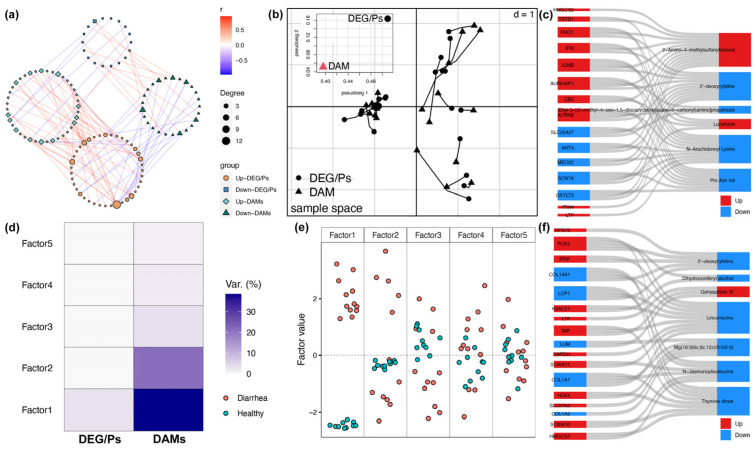
The associations between shared DEG/Ps and DAMs in the colon of piglets. (**a**) The correlation network of shared DEG/Ps and DAMs is based on Spearman’s correlation with *p*-value adjusted by the false detection ratio (FDR) method. It is noteworthy that only correlations that were found to be statistically significant (|r| > 0.8 and FDR < 0.05) are displayed. (**b**) Sample co-variation based on the MCIA and the eigenvalues of shared DEG/Ps and DAMs at the first two pseudoeig axes. (**c**) The Sankey diagram is employed to illustrate the correlations between the expression levels of the top 20 important shared DEGs/Ps and the abundances of the top 20 important DAMs, as identified by the MCIA. It is important to note that only correlations that have been determined to be statistically significant (|r| > 0.8 and FDR < 0.05) are displayed by the grey connecting lines. (**d**) A heatmap is employed to illustrate the explanatory proportion of the top 5 integration factors obtained by the MOFA to the variations in shared DEG/Ps and DAMs. (**e**) Scatter plot illustrating the distribution of factor values of each sample among the top 5 integration factors obtained by the MOFA. (**f**) The Sankey diagram exhibits the correlations between the expression levels of the top 20 important shared DEG/Ps and the abundances of the top 20 important DAMs that were identified by the MOFA. It is noteworthy that only correlations that have been determined to be statistically significant (i.e., those with a magnitude of r greater than 0.8 and a false discovery rate (FDR) less than 0.05) are displayed by the grey connecting lines.

In the colon, a total of 84 links between shared DEGs/Ps and DAMs were identified in the correlation network (Figure 7a and Figure S14). Within this correlation network, five module hubs (three DAMs: LysoPC (P-18:1(9Z/0:0)), N-desmethylritonavir, and paaric acid; two DEG/Ps: RKP3 and METTL26) and six connectors (four DAMs: LysoPC (0:0/20:4(5Z,8Z,11Z,14Z)), arginylproline, N-acetyl-9-aminominocyclin, and Lyso-PC (18:0); and two DEG/Ps: CAMK2B and GEMIN8) were identified (Figure S14c). The first two components of the MCIA explained 71% and 53% of the variation in the number of shared DEGs/Ps and DAMs, respectively (Figure 7b). Furthermore, the top 20 important DEGs/Ps and DAMs identified in the MCIA were significantly correlated with 2-amino-4-methylsulfanybutanal, 2-deoxycytidine, lumiflavin, N-arachidonoyl lysine, and Pro-Asn-Val (Spearman correlation, *p* < 0.05; Figure 7c). Furthermore, the top five integration factors obtained by the MOFA explained 60.49% of the DAM changes but only 3.05% of the shared DEG/P variations (Figure 7d). Utilising the factor values of diverse samples, the top 2 factors were able to differentiate jejunum samples exhibiting distinct health statuses (Figure 7e), and the key DEG/Ps and DAMs for these factors were identified (Figures S15 and S16). Among these biomarkers, the majority of the DEGs/Ps presented significant correlations with 2-deoxycytidine, dihydroconiferyl alcohol, gonyautoxin VI, lincomycine, Mg (18:3(6z,9z,12z)/0:0/0:0), N-jasmonoylisoleucine, and thymine dimer (Spearman correlation, *p* < 0.05; Figure 7f).

## 4. Discussion

Multiomics research is increasingly utilised in livestock or veterinary science to provide a comprehensive view of the host’s interactions with pathogens and environmental factors [34,35]. These insights have the potential to offer valuable parallels for livestock health or enhance our understanding of human health research, particularly in the field of infectious diseases [36]. For example, multiomics approaches have been employed to investigate the impact of different strains of influenza A virus on pigs. In this context, innate immune responses during infection have been characterised via global proteomics and RNA sequencing [28]. Furthermore, the integration of the untargeted metabolome and lipidome with machine learning has enabled the identification of lysophosphatidic acid as a biomarker and therapeutic target for porcine reproductive and respiratory syndrome [6]. Research in this field has also focused on PEDs, including the genetic variations and virulence of PEDV [37,38], immune responses of piglets [1], and potential vaccine targets [39]. Specifically, a previous study identified betaine-homocysteine S-methyltransferase as a plausible intervention target for PED by integrating metabolomic and proteomic data [40]. Continued exploration via integrated omics approaches will be crucial for developing effective management strategies in livestock production systems. To the best of our knowledge, the present study is the first attempt to comprehensively analyse the immune response of piglets during diarrhea by integrating transcriptome, proteome, and metabolome data.

### 4.1. Suspected Viral Infection Activates the Complement Cascade but Inhibits the Complement Receptor Interaction

**Figure 8:**
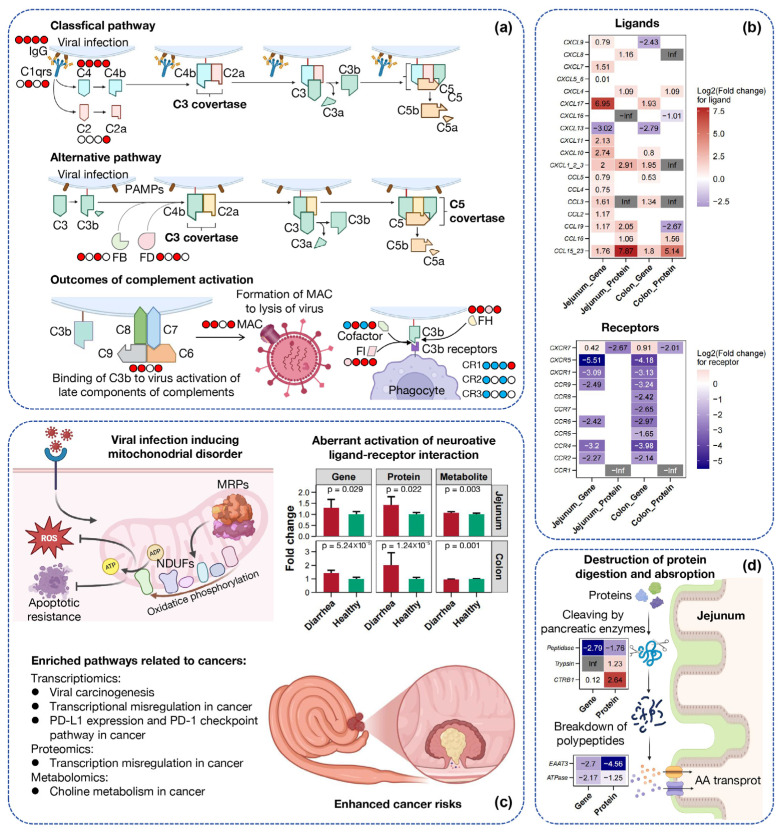
The response mechanisms of piglets to diarrhea due to viral infection. (**a**) The initial phase of the viral infection process involves the activation of the complement cascade, while concurrently, the complement receptor (CR) interaction is inhibited. The adjacent circles represent the differential expression of corresponding genes or proteins in the jejunum of piglets with diarrhea in comparison to healthy counterparts. For the left-to-right orientation, these circles represent the jejunum transcriptomes, jejunum proteomes, colon transcriptomes, and colon proteomes, respectively. The colour coding system utilised in this figure. involves the use of red and blue to denote significantly up- and down-regulated genes, respectively. No significant variation is indicated by white, pink circles indicate genes/proteins that showed no significant change in all four datasets. (**b**) Heatmaps reveal the log2-fold change in chemokines and their receptors between the diarrhea piglets and the healthy samples in different datasets. The value and colour of each block represent the log2-transformed fold change in the corresponding ligand or receptor. Inf and -Inf designate instances where the ligand or receptor was not detected in healthy piglets, while white blocks indicate undetected ligand or receptor in diarrhea piglets. (**c**) The concept diagram illustrates the enhanced immune-related diseases risks in diarrhea piglets due to the mitochondrial disorder and aberrant neuroactive ligand–receptor interaction. The barplot reveals the fold change in expression level of the neuroactive ligand–receptor interaction pathway between diarrhea and healthy piglets. The *p*-value obtained by the Wilcox rank-sum test. (**d**) The concept diagram illustrates the disruption of protein digestion and absorption in the jejunum of piglets due to diarrhea. Heatmaps are employed to reveal the fold change in related functional terms between the diarrhea piglets and the healthy samples in different datasets. The value and colour of each block represent the log2-transformed fold change in the corresponding functional term. The term ‘Inf’ is used to denote functional terms that were not detected in healthy piglets.

The results of the transcriptomic analysis demonstrated that the complement and coagulation cascades pathway was the most significantly enriched pathway in the colons of diarrheic piglets (Figure 1c). A more detailed analysis of the classical and alternative pathways of the complement cascades revealed their activation, as evidenced by the upregulation of nearly all pathway members at both the transcriptional and protein levels in the colons of diarrheic piglets (Figure 8a). The classical complement pathway is primarily initiated by the formation of antigen–antibody complexes involving immunoglobulin G (IgG) antibodies [41]. Viral infections have been shown to stimulate the production of IgG antibodies, which are essential for neutralising viruses and activating various immune responses [42]. In this study, a significant increase in IgG protein expression was observed in the colons of diarrheic piglets (Figure 8a). Although we did not directly confirm viral presence, the upregulation of IgG and complement components is consistent with a viral etiology. The alternative complement pathway is directly triggered by foreign materials and plays a significant role in the early immune response, particularly before specific antibodies are produced [43]. The activation of this pathway can be initiated by the spike protein or pathogen-associated molecular patterns present on the surface of viruses [44]. The terminal phase of the complement pathway involves the formation of the membrane attack complex (MAC), which creates pores in bacterial membranes, leading to cell death [45]. During a viral infection, MACs can form on virus-infected cells or directly on the virion, leading to lysis of the viral envelope and effective neutralisation of the virus [46]. However, the use of MAC alone may not be sufficient to eliminate viral infections in piglets, given the occurrence of severe diarrhea.

In addition to direct action, complement activation enhances adaptive immune responses via the anaphylatoxins C3a and C5a to recruit and activate other immune cells [47]. The functionality of these immune systems is contingent upon the interplay between anaphylatoxins and a variety of complement receptors (CRs) [48]. The expression of nearly all CRs, including CR1-CR4 and CR51R, was downregulated in the colons of diarrheic piglets (Figure 8a). CRs play a significant role in the adaptive immune response by facilitating interactions between innate and adaptive immune cells, enhancing the activation and regulation of B and T lymphocytes [49]. The enhancement of antigen-presenting cells’ ability to recognise, internalise, and present antigens, a crucial step in T-cell activation [50], is facilitated by some CRs. Furthermore, when B cells engage with complement-opsonised antigens, they receive additional signals that promote their activation and differentiation into antibody-secreting plasma cells [45]. Deficiencies in these receptors have been linked to increased susceptibility to infections or autoimmune diseases [51,52], which is consistent with the enrichment of pathways involving virus infections and immune diseases in the colons of diarrheic piglets (Figure 1c).

### 4.2. Suspected Viral Infection Induces the Secretion of Chemokines but Limits Their Immune Signal Transduction During Diarrhea in Piglets

The pathways related to cytokine–cytokine receptor interactions were enriched in both the jejunum and the colon of piglets with diarrhea, as indicated by both the DEGs and DEPs (Figure 4c). Cytokines are defined as essential signalling molecules that are typically soluble proteins or glycoproteins released by cells in response to stimuli [53]. They bind to specific receptors on target cells to elicit a biological response, particularly in immune responses, inflammation, and various physiological processes [54]. Among the diverse array of cytokines, the ligands of chemokines, encompassing the CC and CXC subfamilies, exhibited consistent upregulation at both the transcriptional and protein levels in the jejunum and colon of diarrheic piglets (Figure 8b). This phenomenon aligns with the previous common discovery that viral infections can indeed induce the production of chemokines [55,56]. The function of chemokines is to guide leukocytes to sites of infection through a process known as chemotaxis. Once they are present, they bind to their receptors on target cells, triggering a series of host defense mechanisms, including cell adhesion and the production of inflammatory molecules [57]. The process of ligand-mediated dimerisation with receptors is imperative for the activation of intracellular signalling cascades that are mediated by cytokines [58]. However, in contrast to the induction of chemokines, the levels of their receptors consistently decreased due to diarrhea (Figure 8b). The absence of chemokine receptors has been shown to disrupt the cascade, resulting in impaired signalling and subsequent immune dysfunction [59,60]. The redundancy of chemokines can lead to various clinical manifestations ranging from mild immunodeficiency to severe systemic inflammatory responses [61], which is consistent with the significant enrichment of various pathways related to the immune system and diseases found in pigs with diarrhea (Figure 4c).

### 4.3. Diarrhea in Piglets Is Associated with Increased Immune-Related Diseases Risk Due to Mitochondrial Disorders and Aberrant Neuroactive Ligand–Receptor Interactions

MRPs and NDUFs represent critical components of mitochondrial function, particularly in the context of energy metabolism and the regulation of cellular processes [62]. MRPs have been shown to contribute to the assembly of mitochondrial ribosomes that synthesise proteins essential for NDUFs [63]. In addition to their role in protein synthesis, the aberrant expression of MRPs and NDUFs has been linked to apoptosis and immune-related diseases [64,65]. Aberrant expression of MRPs can lead to a reliance on oxidative phosphorylation, which some disease types exploit for survival under nutrient-limited conditions [66]. Furthermore, the upregulation of MRPs has been associated with the modulation of the mitochondrial pathway of apoptosis, with the promotion of apoptotic resistance facilitating tumor growth and metastasis [67]. The expression levels of MRPs correlate with clinical features such as prognosis and metastasis in various immune-related diseases; thus, MRPs are considered potential biomarkers for infectious disease diagnosis [68]. The upregulated expression of NDUF subunits, particularly NDUFB3 and NDUFC1, has been identified as a crucial regulatory factor in the mitochondrial pathway of apoptosis and in the production of reactive oxygen species [69,70]. These processes are integral to the survival and proliferation of immune cells. Exploration is underway for new small-molecule inhibitors targeting components of NDUF, with the potential to inhibit oxidative phosphorylation in diseases [71]. The analysis of mitochondrial function in jejunum and colon samples revealed a high diseases risk in diarrheic piglets (Figure 8c). Further investigations into MRPs may reveal potential therapeutic intervention pathways.

Another signal transduction pathway, namely, the neuroactive ligand–receptor interaction pathway, was enriched in diarrheic piglets according to all the omics datasets in this study (Figure 4c and Figure 5d). The neuroactive ligand–receptor interaction pathway is imperative for the mediation of intracellular and extracellular signalling processes, which in turn influence various biological functions, including pain perception and immune responses [72]. Moreover, recent studies have underscored the pivotal role of this pathway in immune-related disease. For example, it has been shown to be overactivated in tumours with homologous recombination deficiency, contributing to immunosuppression in colon cancer [73]. Furthermore, this pathway has been identified as being significantly upregulated in human subjects diagnosed with lung and prostate related disease [74,75]. Despite the identification of altered levels of multiple pathway members in diarrheic piglets, a comprehensive analysis across all three aspects (transcriptomic, proteomic, and metabolomic) revealed a marked upregulation of this pathway in diarrheic piglets compared with their healthy counterparts (Figure 8c). In conjunction with the enriched immune-related pathways observed in the colon of piglets with diarrhea (Figure 4c and Figure 5d), these findings suggest a heightened immune-related disease risk, particularly in the colon, in piglets with diarrhea (Figure 8c).

Furthermore, a better understanding of neuroactive ligand–receptor interactions has the potential to yield novel insights, paving the way for the development of patient-tailored therapies and the enhancement of immunotherapeutic strategies, with the ultimate aim of improving patient outcomes. Specific genes within this pathway, such as LTB4R2, have been identified as novel targets for therapeutic intervention, suggesting that inhibiting this pathway could increase the efficacy of immunotherapy in colon cancer patients [73]. Furthermore, the downregulation of calcitonin receptor (CALCR) has been associated with increased glioblastoma growth, and targeting CALCR could provide a therapeutic avenue for improving patient outcomes [76]. Furthermore, functional analyses have indicated that targeting components of this signalling pathway may be beneficial for the treatment of pancreatic disease [77]. In this study, galanin (GAL), trypsin (PARRS), and anandamide should be given more attention because of their increased abundance in both the jejunum and the colon of diarrhea-afflicted piglets (Figure 8c). Galanin, a neuropeptide encoded by the GAL gene, is widely expressed in the gastrointestinal tract of mammals [78]. Galanin is considered a potential target for treating diabetes because of its regulatory effects on insulin secretion [79]. Trypsin, a crucial serine protease enzyme, is produced by the pancreas and plays a significant role in the digestion of proteins in the small intestine [80]. In the context of therapeutic applications, trypsin is employed topically to facilitate the removal of necrotic tissue from wounds and promote the healthy growth of surrounding tissue [81]. Anandamide, a significant fatty acid neurotransmitter, plays a crucial role in the endocannabinoid system [82]. It has been demonstrated to induce apoptotic cell death by activating caspase-3 [83]. The elevated levels of this substance in piglets, concomitant with increased disease risk, underscore its potential as a therapeutic target. However, further research is imperative to elucidate the precise mechanisms underlying its function.

### 4.4. Destruction of Protein Digestion and Absorption During Diarrhea in Piglets

During the period of early-life development (PED) in piglets, porcine epidemic diarrhea virus (PEDV) replicates in the enterocytes of the small intestine, leading to impaired absorption of fluids and nutrients, resulting in severe diarrhea and dehydration [84]. While we did not perform direct pathogen detection in this study, our observed molecular changes (e.g., trypsin increase, peptidase reduction) are consistent with PEDV-induced pathology. The protein digestion and absorption pathway was significantly enriched in the jejunum of diarrheic piglets on the basis of both DEGs and DEPs (Figure 4c). The digestion and absorption of proteins primarily occur in the duodenum and proximal jejunum of the small intestine, following initial digestion in the stomach [85]. Once chyme enters the small intestine, further digestion occurs through the action of pancreatic enzymes [86]. These enzymes function by cleaving peptide bonds within polypeptides, thereby breaking them down into smaller peptides and eventually into tripeptides, dipeptides, and free amino acids [87]. In this study, we did not observe significant downregulation of pancreatic proteases in the jejunum of diarrheic piglets, and one of them, chymotrypsinogen B1 and trypsin, was more abundant (Figure 8c,d). These results suggested that diarrhea could not disturb the digestion of proteins mediated by pancreatic enzymes. However, the metabolomic analysis indicated significant accumulation of polypeptides, particularly Asn-Leu-Ser-Pro-Asn, in the jejunum of diarrheic piglets (Figure 6c,f), which implies potential negative effects of diarrhea on the downstream processes of polypeptide digestion and absorption.

Following the initial breakdown of proteins by pancreatic enzymes, the action of peptidases located on the microvilli of intestinal epithelial cells facilitates protein digestion, thereby completing the breakdown of polypeptides into individual amino acids [88]. The transcript and protein levels of peptidase were both significantly downregulated in the jejunum of diarrheic piglets, indicating a reduction in peptide breakdown during diarrhea (Figure 8d). During the absorption of amino acids in the small intestine, some amino acids are absorbed through specific transporters that do not require energy [89]. Notably, excitatory amino acid transporter 3 (EAAT3) was downregulated in the jejunum of diarrheic piglets at both the transcriptional and protein levels (Figure 8d). EAAT3 plays a pivotal role in protein digestion by facilitating the transport of essential amino acids, such as cysteine and glutamate, into intestinal epithelial cells [90]. Its function extends beyond mere transport, as it also influences cellular processes through the activation of the mTOR pathway, thereby linking nutrient availability to cell growth and metabolism [91]. Alternatively, the absorption of amino acids by the small intestine frequently involves sodium-dependent transporters that utilize ATP to transport amino acids against their concentration gradient into intestinal cells [92]. This process is particularly dependent on the Na^+^, K^+^-ATPase located in the basolateral membrane of intestinal epithelial cells [93]. The Na^+^-dependent transporters responsible for the absorption of amino acids and peptides from digested proteins are supported in their function by Na^+^-dependent transporters [94]. As anticipated, the expression levels of Na^+^, K^+^-ATPase, as determined by both transcriptomic and proteomic datasets, were markedly reduced in the jejunum of diarrheic piglets (Figure 8d). Consequently, a comprehensive and systematic breakdown in polypeptide digestion and absorption was observed in the jejunum of diarrheic piglets (Figure 8d).

### 4.5. Limitations

Several limitations should be acknowledged. First, we did not perform direct pathogen detection (e.g., PCR for PEDV, bacterial culture), so the attribution to viral infection is inferential. Second, the sample size was not based on a formal power calculation, though it is comparable to similar multi-omics studies. Future studies with larger cohorts are warranted to validate our findings. Third, functional validation (e.g., gene knockout or inhibitor studies) was not performed, so causality remains to be established. In addition, individual sow identities and litter assignments were not recorded during sample collection, which prevented us from statistically controlling for potential litter effects (e.g., including litter as a random effect). Nevertheless, the balanced group sizes (*n* = 20 per group) and the large effect sizes observed suggest that any undetected litter effect is unlikely to overturn the main conclusions. Future studies should record litter information and incorporate litter as a random effect to increase statistical rigor.

## 5. Conclusions

In summary, the present study investigated the underlying responses of newborn using piglets model during diarrhea via a multiomics approach. First, diarrhea in piglets disrupts the digestion and absorption of nutrients, particularly protein, in the jejunum, resulting in the accumulation of polypeptides in chyme entering the small intestine. Second, the study revealed that the complement system in the colon of piglets was activated by viral infection during diarrhea. However, the infection was not eliminated due to the inability of CRs to activate adaptive immune functions. Third, the study revealed that the induction of chemokines in the jejunum and colon of piglets due to diarrhea was accompanied by a reduction in their receptor expression, thereby impeding immune signal transduction and contributing to the development of severe immune diseases. Fourth, the overexpression of MRPs and NDUFs, in combination with the hyperactive neuroactive ligand–receptor interactions observed in both the jejunum and colon of diarrheic piglets, suggested a high intestinal immune disease risk. These findings contribute to our expanded understanding of PED and identify potential therapeutic targets for intervention for newborn or young animals. Future research should focus on: (i) functional validation of MRP and NDUF overexpression using knockout or inhibitor models; (ii) testing nutritional interventions that enhance peptidase activity or restore amino acid transporter expression; and (iii) conducting longitudinal studies to capture dynamic changes during disease progression.

## Data Availability

Data are available from the authors upon request.

## Supplementary Materials

The following supporting information can be downloaded at: https://www.mdpi.com/article/10.3390/ani16111671/s1, Figure S1: Gene expression profiles associated pathways in piglets based on the KEGG database; Figure S2: Numbers of DEGs identified in jejunum and colon of piglets belonged to different functions based on the KEGG database; Figure S3: Numbers of up- and down-regulatedly expressed transcriptional factors in Jejunum and colon of piglets with diarrhea compared to the healthy counterparts; Figure S4: Clustering of DEGs identified from the jejunum and colon piglets based on the WGCNA; Figure S5: Protein expression profiles associated pathways in piglets based on the KEGG database; Figure S6: Numbers of DEPs identified in jejunum and colon of piglets belonged to different functions based on the KEGG database; Figure S7: (a) Venn diagram for detected transcripts of RNA-Seq and proteins of protome. (b) Mantel test and Procrustes analysis revealing the correlation between the gene and protein expression profiles among all studied samples. Nine quadrant diagrams for the variations in the detected proteins with their coding genes in jejunum (c) and colon (d), respectively, between diarrhea and healthy piglets; Figure S8: The eigenvalues of DEGs and DEPs identified from jejunum and colon, respectively, at the first two pesudoeig axes obtained by the MCIA; Figure S9: Numbers of DAMs belonged to different compound classes based on the HMDB identified in Jejunum and colon of piglets with diarrhea compared to the healthy counterparts; Figure S10: Numbers of DAMs identified in jejunum and colon of piglets belonged to different functions based on the KEGG database; Figure S11: Numbers of nodes (a) and edges (b) in the correlation network of shared DEG/Ps and DAMs identified in jejunum of diarrhea piglets compared to healthy controls. (c) Zi-Pi plot showing the distribution of shared DEG/Ps and DAMs based on their topological roles. The threshold values of Zi and Pi used for categorising were 2.5 and 0.62, respectively; Figure S12: Absolute loadings for the DEG/Ps with the largest absolute weights in the jejunum proteome data for top 2 factors obtained by the MOFA. Plus or minus symbols on the right indicate the sign of the loading; Figure S13: Absolute loadings for the DAMs with the largest absolute weights in the jejunum metabolome data for top 2 factors obtained by the MOFA. Plus or minus symbols on the right indicate the sign of the loading; Figure S14: Numbers of nodes (a) and edges (b) in the correlation network of shared DEG/Ps and DAMs identified in colon of diarrhea piglets compared to healthy controls. (c) Zi-Pi plot showing the distribution of shared DEG/Ps and DAMs based on their topological roles. The threshold values of Zi and Pi used for categorising were 2.5 and 0.62, respectively; Figure S15: Absolute loadings for the DEG/Ps with the largest absolute weights in the colon proteome data for top 2 factors obtained by the MOFA. Plus or minus symbols on the right indicate the sign of the loading; Figure S16: Absolute loadings for the DAMs with the largest absolute weights in the colon metabolome data for top 2 factors obtained by the MOFA. Plus or minus symbols on the right indicate the sign of the loading.
