# Supplementary material for: Transcriptome, Proteome, and Metabolome Features in Diarrhea Challenge of Preweaning Piglets via Multi-Omics Integration Analyses
Source: Animals (Basel). 2026 May 29;16(11):1671. doi: 10.3390/ani16111671 (PMC13255689; doi:10.3390/ani16111671)
Supplement: Supplementary file 1 [file animals-16-01671-s001.zip › Supplementary Materials.pdf]

Transcriptome, Proteome, and Metabolome Features in Diarrhea Challenge of Preweaning Piglets Via Multi-omics Integration Analyses

Shilong Zhao[1], Siyi Peng[1], Guangxin Yang[1], Haitao Yu[1,*] and Shiyan Qiao[1]

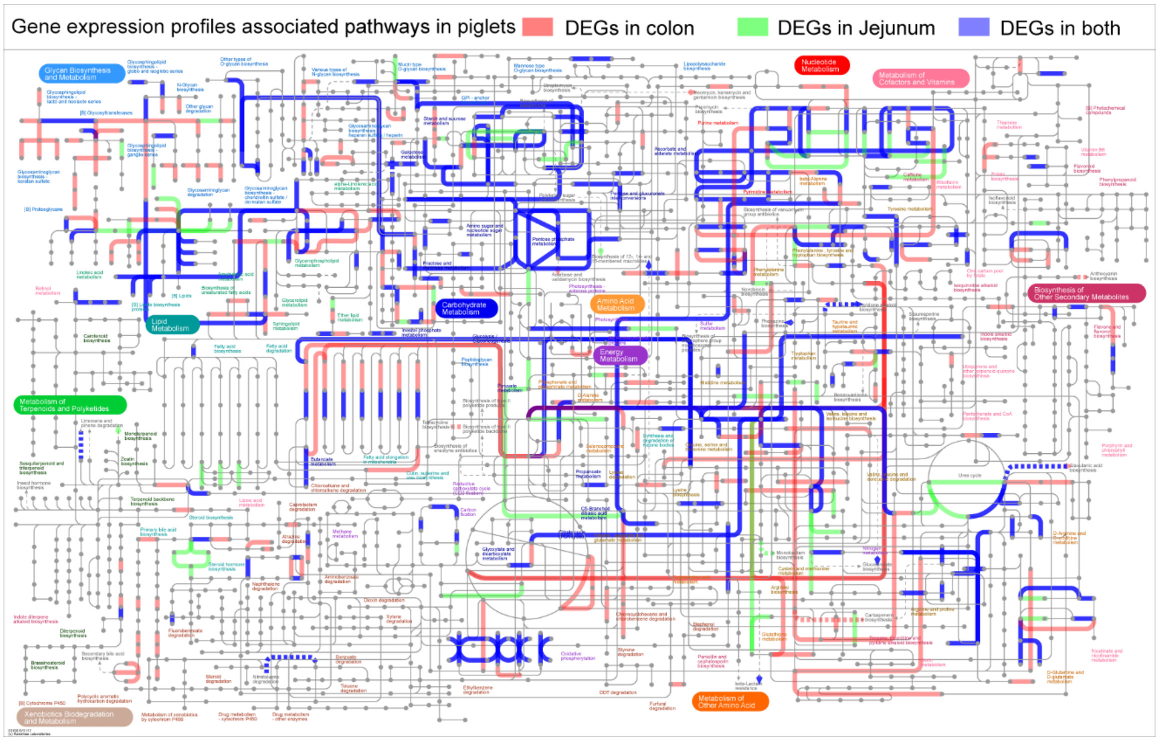

Figure S1. **Gene expression profiles associated pathways in piglets based on the KEGG database.**

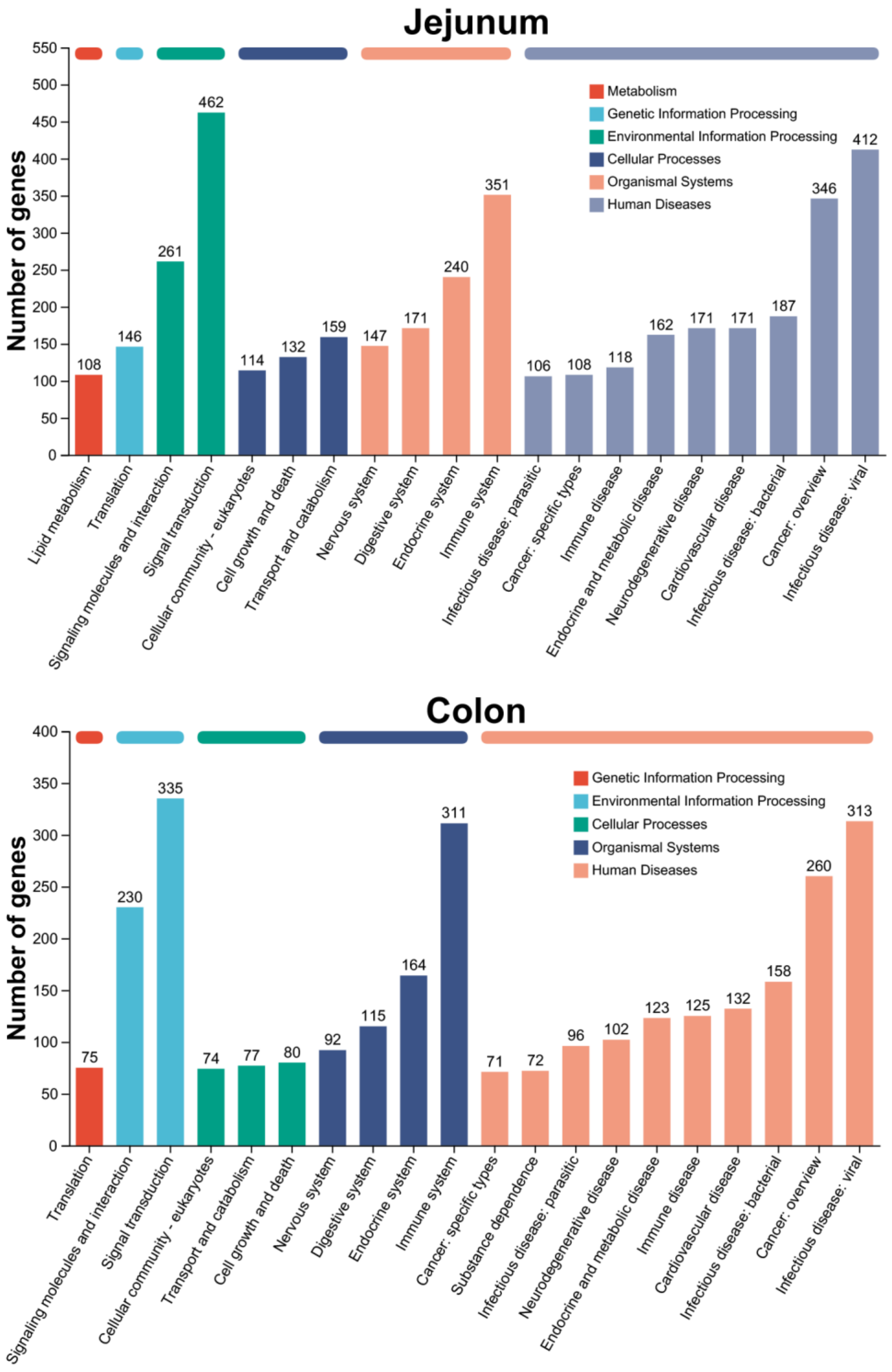

Figure S2. **Numbers of DEGs identified in jejunum and colon of piglets belonged to different functions based on the KEGG database.**

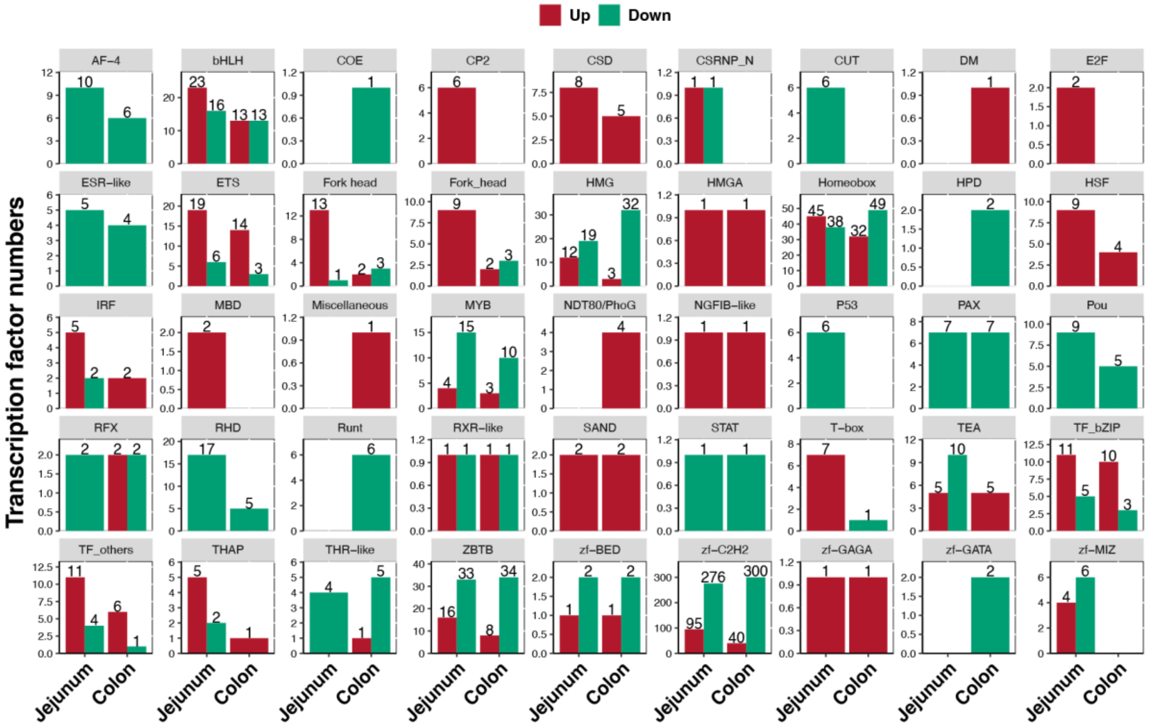

Figure S3. **Numbers of up- and down-regulatedly expressed transcriptional factors in Jejunum and colon of piglets with diarrhea compared to the healthy counterparts.**

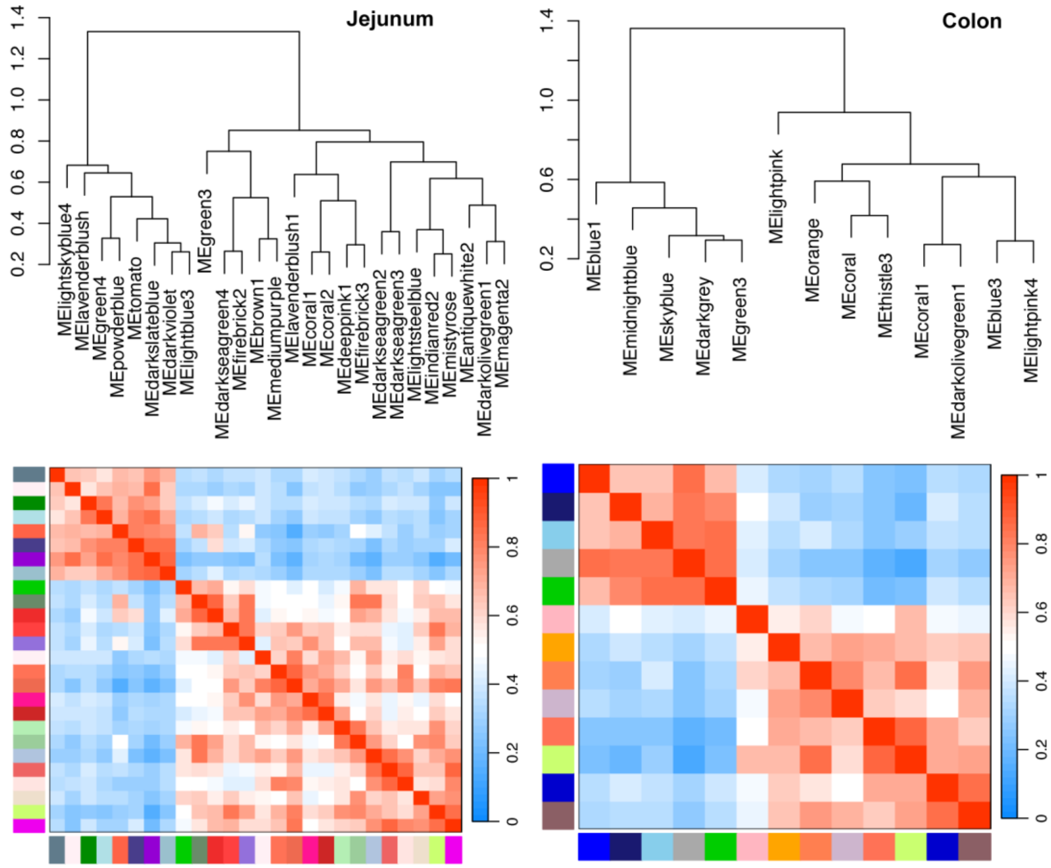

Figure S4. **Clustering of DEGs identified from the jejunum and colon piglets based on the WGCNA.**

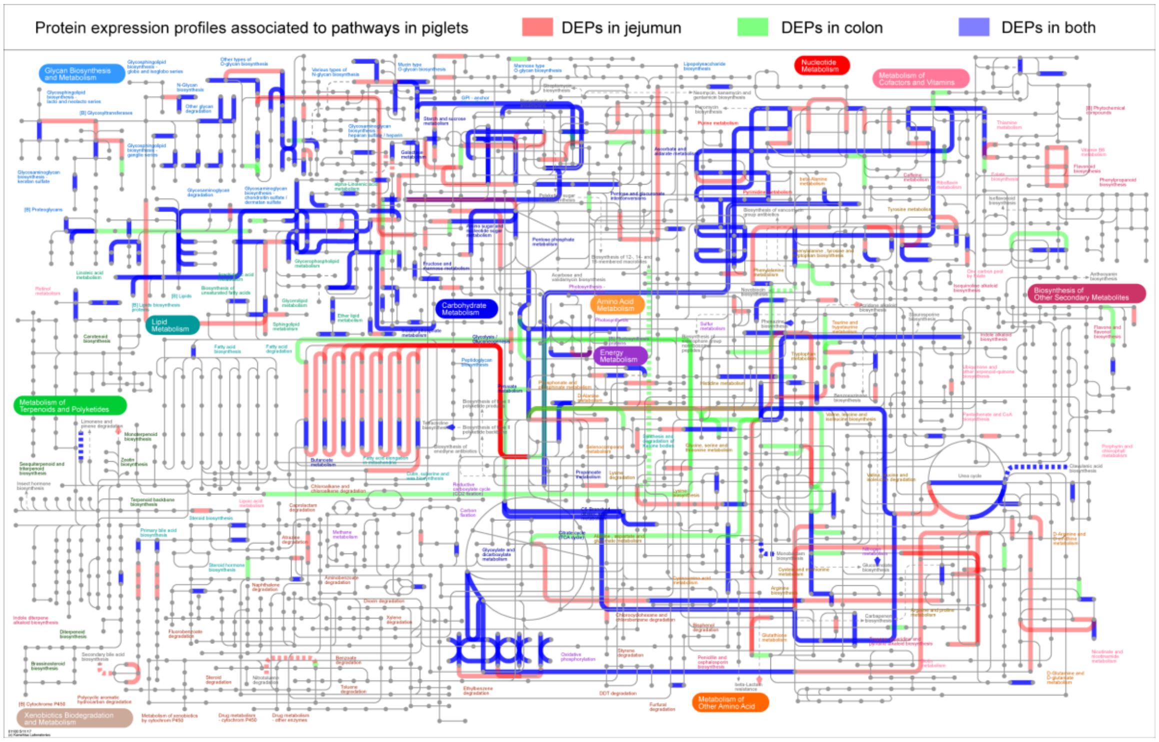

Figure S5. **Protein expression profiles associated pathways in piglets based on the KEGG database.**

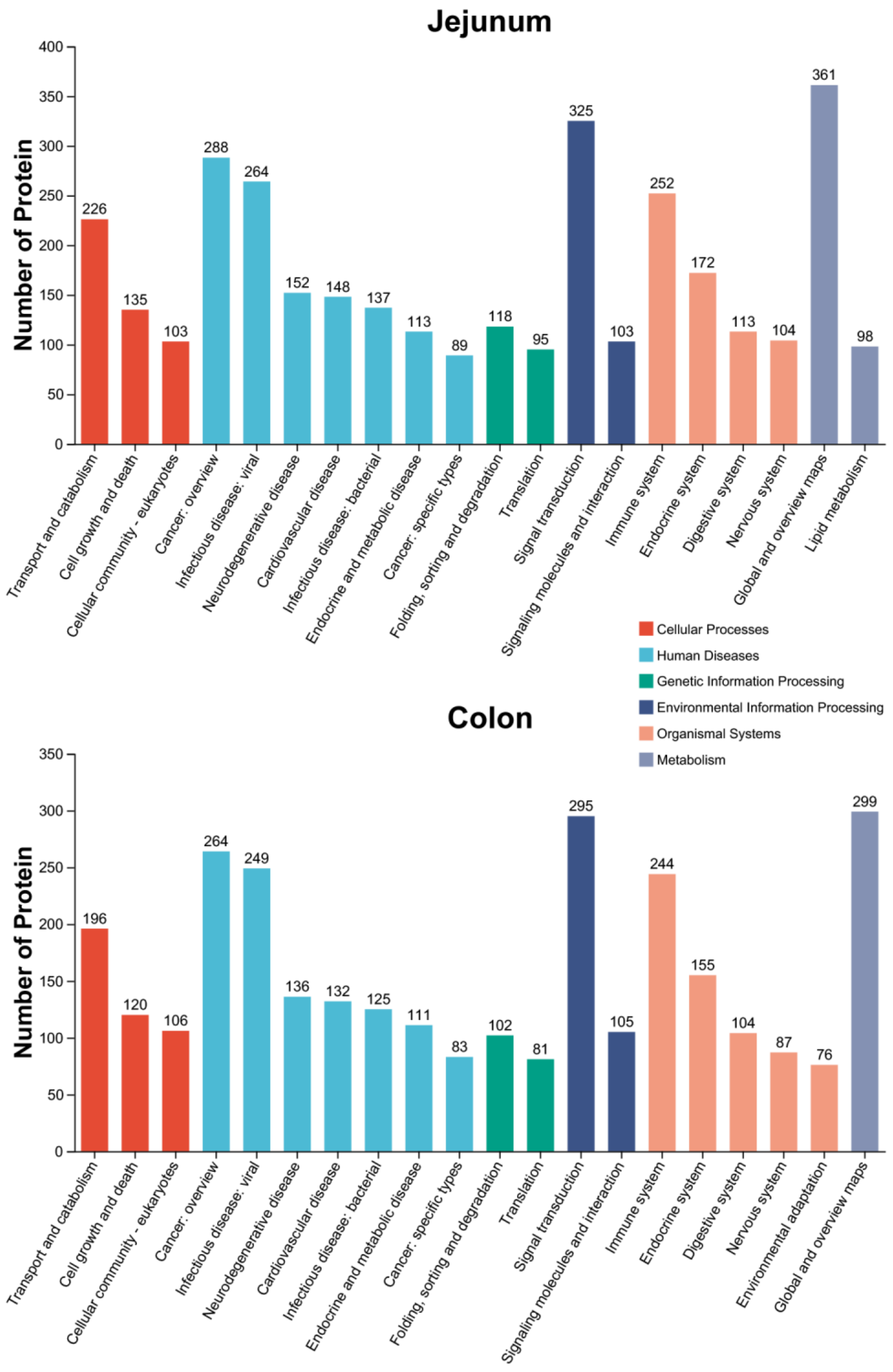

Figure S6. **Numbers of DEPs identified in jejunum and colon of piglets belonged to different functions based on the KEGG database.**

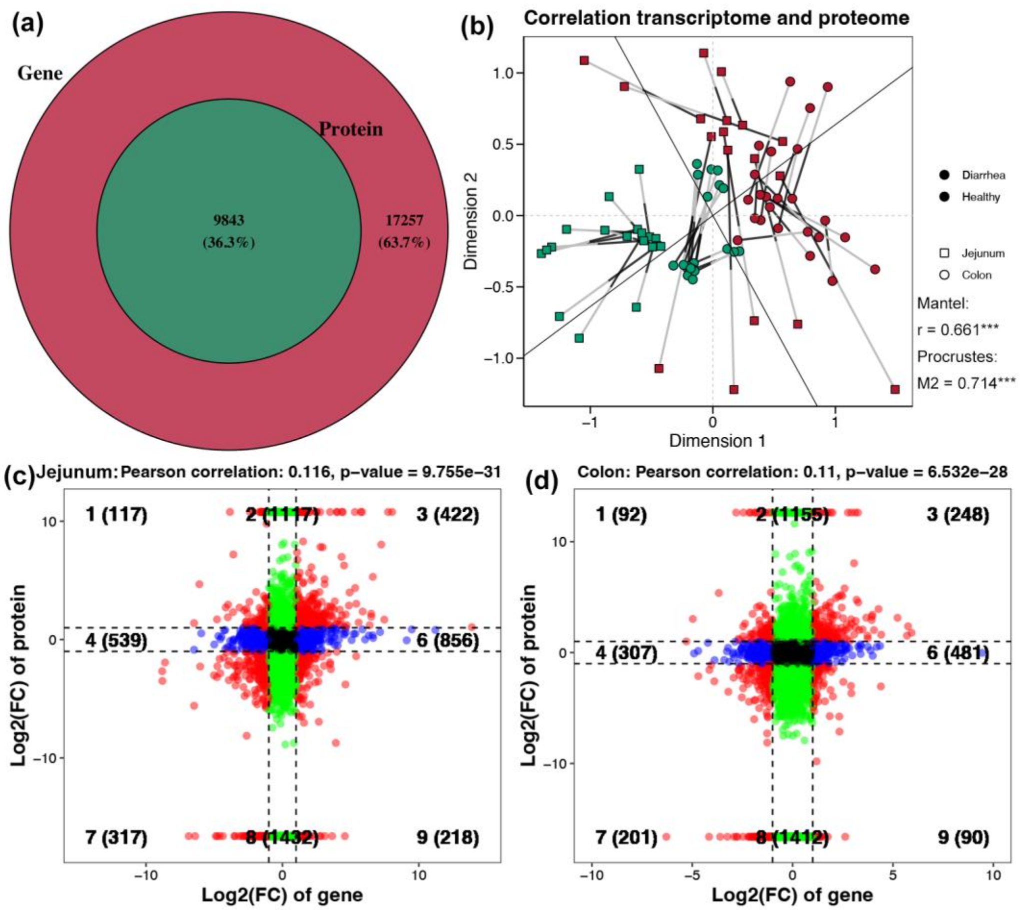

Figure S7. **(a) Venn diagram for detected transcripts of RNA-Seq and proteins of protome. (b) Mantel test and Procrustes analysis revealing the correlation between the gene and protein expression profiles among all studied samples. Nine quadrant diagrams for the variations in the detected proteins with their coding genes in jejunum (c) and colon (d), respectively, between diarrhea and healthy piglets.**

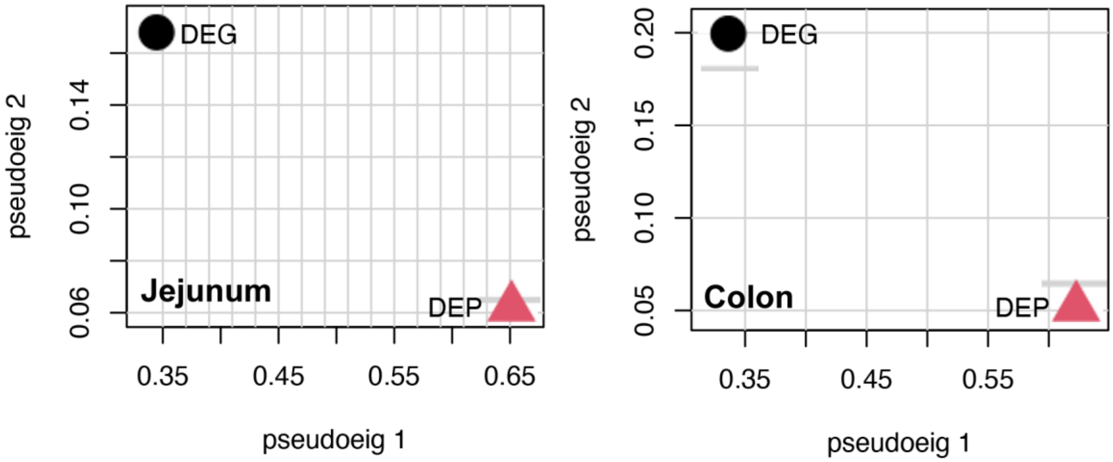

Figure S8. **The eigenvalues of DEGs and DEPs identified from jejunum and colon, respectively, at the first two pesudoeig axes obtained by the MCIA.**

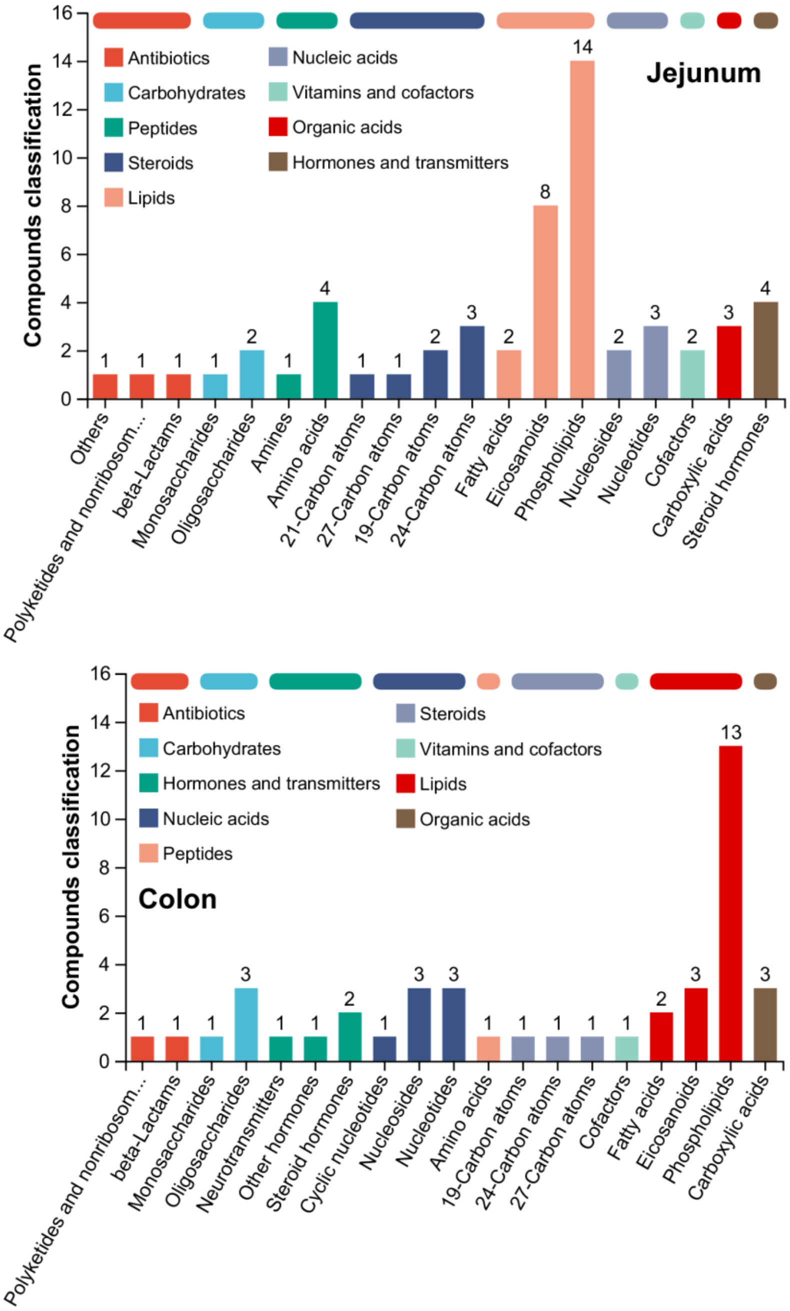

Figure S9. **Numbers of DAMs belonged to different compound classes based on the HMDB identified in Jejunum and colon of piglets with diarrhea compared to the healthy counterparts.**

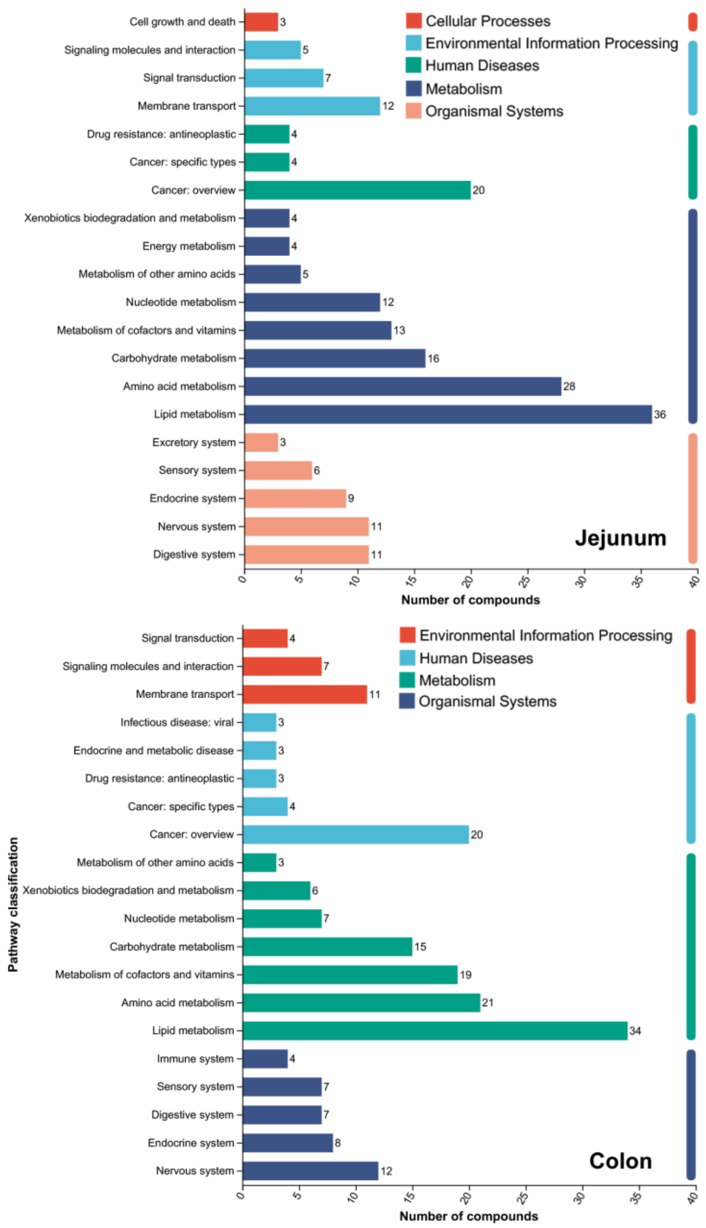

Figure S10. **Numbers of DAMs identified in jejunum and colon of piglets belonged to different functions based on the KEGG database.**

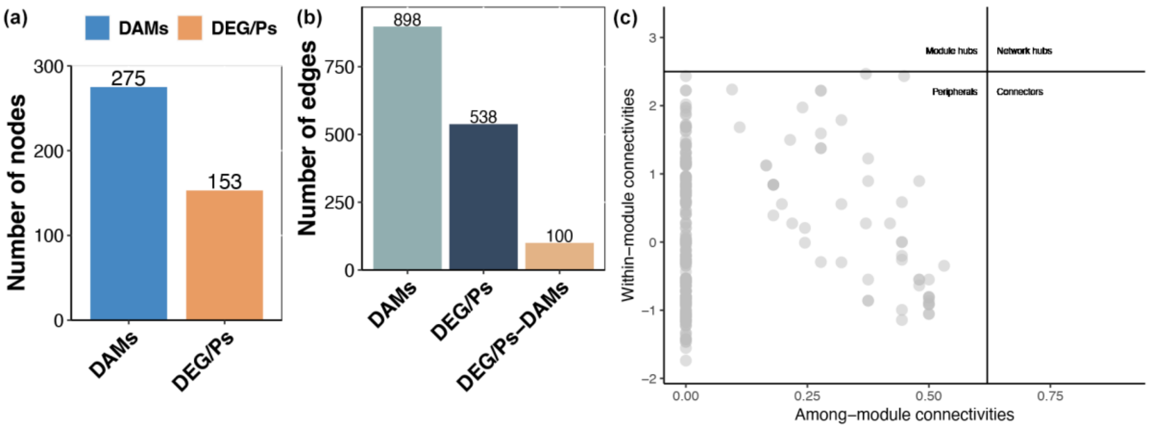

Figure S11. **Numbers of nodes (a) and edges (b) in the correlation network of shared DEG/Ps and DAMs identified in jejunum of diarrhea piglets compared to healthy controls. (c) Zi-Pi plot showing the distribution of shared DEG/Ps and DAMs based on their topological roles. The threshold values of Zi and Pi used for categorising were 2.5 and 0.62, respectively.**

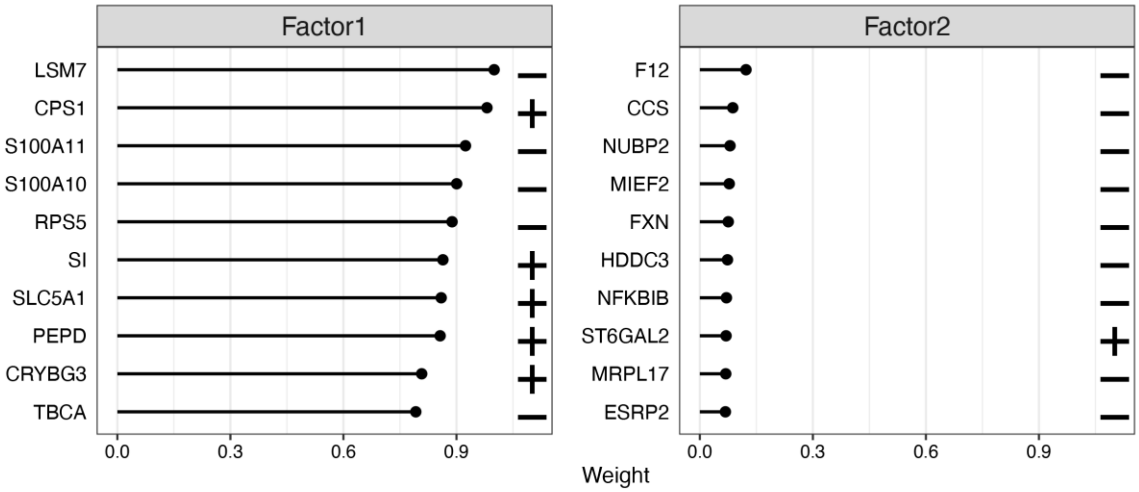

Figure S12. **Absolute loadings for the DEG/Ps with the largest absolute weights in the jejunum proteome data for top 2 factors obtained by the MOFA. Plus or minus symbols on the right indicate the sign of the loading.**

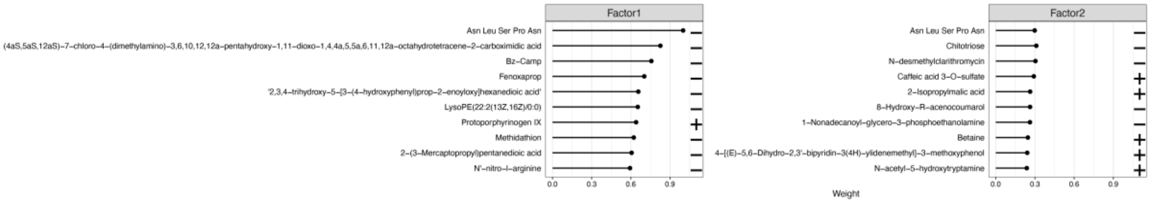

Figure S13. **Absolute loadings for the DAMs with the largest absolute weights in the jejunum metabolome data for top 2 factors obtained by the MOFA. Plus or minus symbols on the right indicate the sign of the loading.**

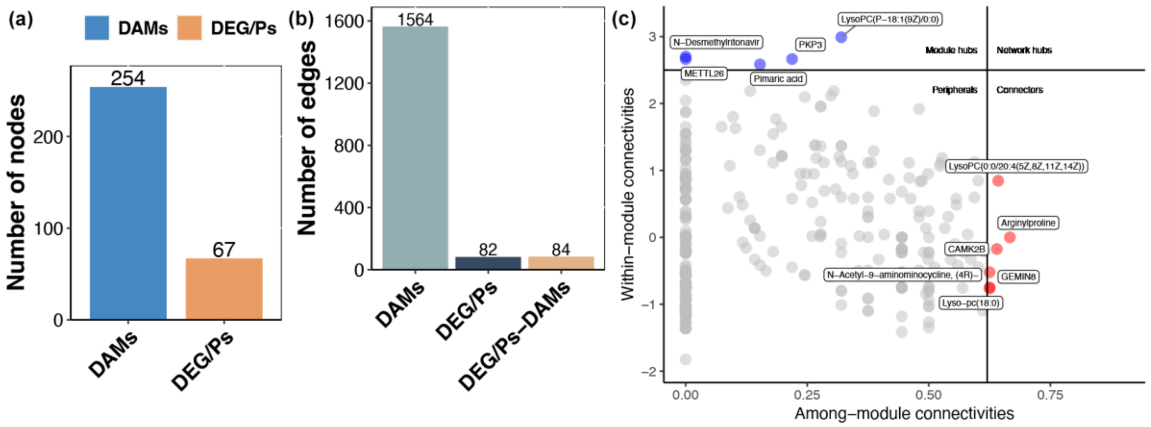

Figure S14. **Numbers of nodes (a) and edges (b) in the correlation network of shared DEG/Ps and DAMs identified in colon of diarrhea piglets compared to healthy controls. (c) Zi-Pi plot showing the distribution of shared DEG/Ps and DAMs based on their topological roles. The threshold values of Zi and Pi used for categorising were 2.5 and 0.62, respectively.**

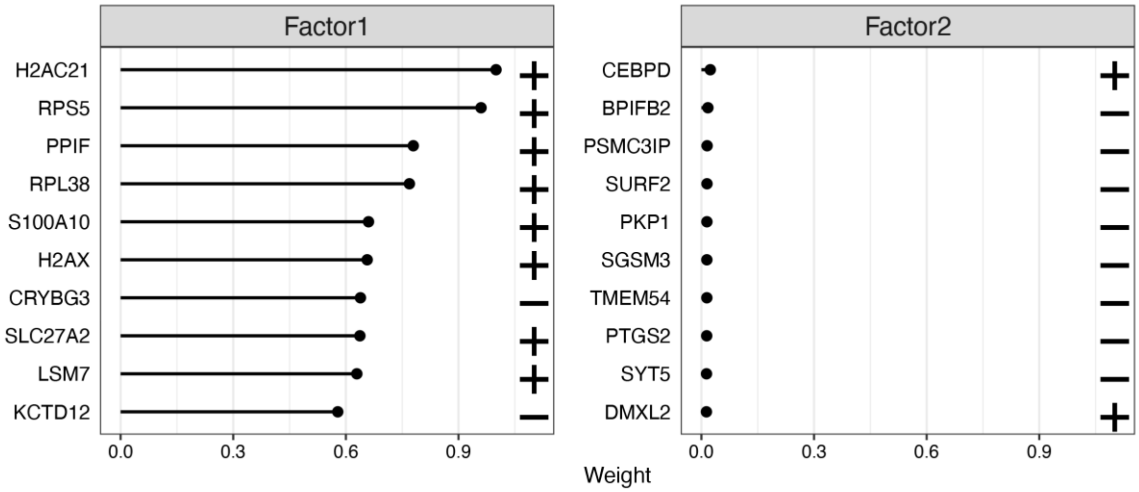

Figure S15. **Absolute loadings for the DEG/Ps with the largest absolute weights in the colon proteome data for top 2 factors obtained by the MOFA. Plus or minus symbols on the right indicate the sign of the loading.**

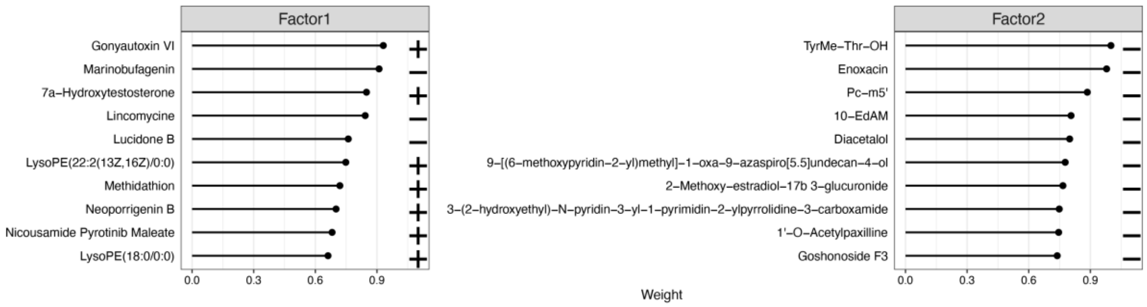

Figure S16. **Absolute loadings for the DAMs with the largest absolute weights in the colon metabolome data for top 2 factors obtained by the MOFA. Plus or minus symbols on the right indicate the sign of the loading.**
